# Pan-cancer landscape of epigenetic factor expression predicts tumor outcome

**DOI:** 10.1038/s42003-023-05459-w

**Published:** 2023-11-16

**Authors:** Michael W. Cheng, Mithun Mitra, Hilary A. Coller

**Affiliations:** 1 Bioinformatics Interdepartmental Program, University of California, Los Angeles, CA USA; 2 Department of Molecular, Cell and Developmental Biology, University of California, Los Angeles, CA USA; 3 Department of Biological Chemistry, David Geffen School of Medicine, University of California, Los Angeles, CA USA; 4 Molecular Biology Institute, University of California, Los Angeles, CA USA

**Keywords:** Cancer genomics, Machine learning

## Abstract

Oncogenic pathways that drive cancer progression reflect both genetic changes and epigenetic regulation. Here we stratified primary tumors from each of 24 TCGA adult cancer types based on the gene expression patterns of epigenetic factors (epifactors). The tumors for five cancer types (ACC, KIRC, LGG, LIHC, and LUAD) separated into two robust clusters that were better than grade or epithelial-to-mesenchymal transition in predicting clinical outcomes. The majority of epifactors that drove the clustering were also individually prognostic. A pan-cancer machine learning model deploying epifactor expression data for these five cancer types successfully separated the patients into poor and better outcome groups. Single-cell analysis of adult and pediatric tumors revealed that expression patterns associated with poor or worse outcomes were present in individual cells within tumors. Our study provides an epigenetic map of cancer types and lays a foundation for discovering pan-cancer targetable epifactors.

## Abstract (web summary)

A pan-cancer machine learning model using epifactor expression data for five cancer types and single-cell analysis of adult and pediatric tumors reveal expression patterns associated with poor or worse outcomes are present in individual cells within tumors.

## Introduction

Epigenetics refers to protein factors and processes that allow the establishment and maintenance of different states, including states of gene activity, at the same genomic locus^1^. Epigenetic processes include changes in DNA methylation, modifications of histone proteins, chromatin accessibility, and higher order chromatin architecture. These changes in state are mediated by chromatin-associated protein factors (epigenetic factors or epifactors) such as those that add, remove, and read DNA and histone modifications, and remodel the chromatin^2^.

Cancer has been historically considered to be a genetic disease with driver mutations in oncogenes or tumor suppressors, but in the last decade, the advent of next generation sequencing technologies has led to a greater appreciation of the role of impaired epigenetic processes, such as inactivation of tumor suppressor genes by promoter DNA methylation, disruption of the regulatory language coded by histone modifications, and aberrant chromatin organization, in cancer progression and drug resistance^3–6^. Genetic mutations and epigenetic changes can have a cooperative effect on cancer development, and one may predispose cancer cells to the other^7–10^. For example, epigenetic silencing of DNA repair genes can lead to new mutations^8^, and compact heterochromatin regions marked by H3K9me3 and H4K20me3 histone marks may be more resistant to new mutations compared to more open euchromatin regions^9^.

Epifactors themselves can be genetically altered in tumors, which can cause widespread epigenetic dysregulation^5,8,11,12^. Mutations in genes involved in DNA methylation and chromatin remodeling are consistently observed in hematological malignancies and solid tumors, respectively^12,13^. Cancer cells can also acquire epigenetic changes in the absence of mutations in epifactor genes^14^. Abnormal expression or repression of epifactor genes in cancer cells can result in epigenetic changes that select for cancer cells with enhanced fitness, resulting in cancer growth^15^. This “non-mutational epigenetic reprogramming” has been proposed as a cancer hallmark^15^, and is thought to be especially important for cancers with few mutations, such as pediatric cancers^12^.

Genomic profiling has shown that tumors from different patients with the same cancer type display intertumor epigenetic heterogeneity^13,16,17^. Understanding epigenetic heterogeneity between patient tumors can provide valuable information about the factors that contribute to variable clinical outcomes and drug responses^18^. Previous studies reporting tumor-to-tumor epigenetic heterogeneity focused mainly on a single epigenetic process such as DNA methylation or chromatin accessibility^9,13,17,19,20^. Because multimodal datasets in which many epigenetic markers are monitored for the same set of tumors are rare due to limited multiplexing of technologies and high cost, a more complete picture of epigenetic differences across tumors is missing. The gene expression levels of epifactors that constitute the epigenetic landscape in tumors are readily available for large pan-cancer patient cohorts (for example, through The Cancer Genome Atlas (TCGA) program). Previous studies on epifactors, however, did not address the relationship between their joint or individual expression patterns and clinical outcomes in detail^10,21^.

In this pan-cancer study, we investigated epigenetic heterogeneity across high purity, primary TCGA patient tumors from each of 24 different adult cancer types by comparing the expression of epifactors. By analyzing the power of epifactors to determine patient outcome across 24 cancer types, we gained analytical breadth to discover cross-tissue patterns and emerging themes that would be missed by focusing on one or a few cancer types^16,21,22^. We found that the expression levels of these epifactors allowed us to classify the patient tumors for each tissue type into distinct clusters with well-defined epifactor signatures. For each tumor type, we asked, How do the clusters relate to clinical outcome? What epifactor signatures that define the clusters are common across the cancer types? What clinically relevant pathways could these epifactors regulate? Does the prognostic value of individual epifactors differ in different cancer types? Do the expression changes in epifactors relate to genetic changes? How does the epifactor landscape of adult tumors compare with pediatric tumors? Finally, what is the relationship between intertumor and intra-tumor (cell-to-cell) heterogeneity? Our study delineates the differences in epigenetic characteristics within and across cancer types through the lens of epifactor expression.

## Results

### Tumors from 24 adult tissue types separate into two distinct clusters based on expression of epifactor genes

**Fig. 1:**
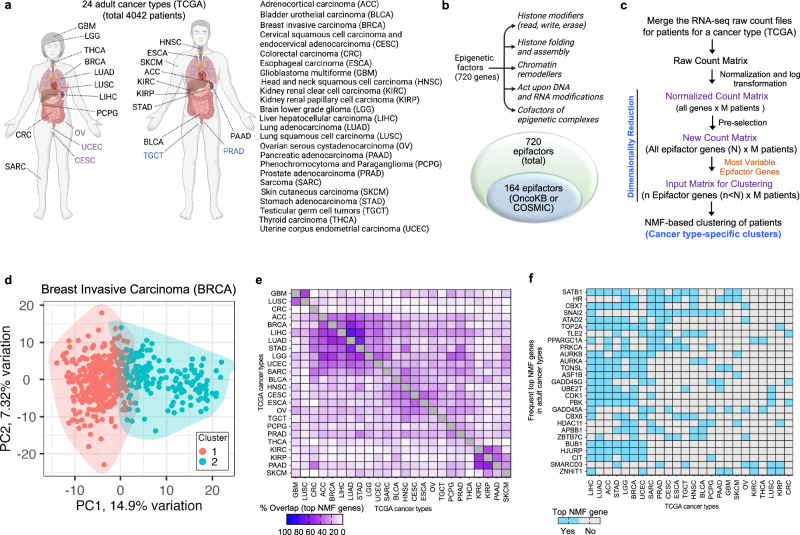
Expression levels of epifactors create two distinct clusters for 24 TCGA cancer types. **a** Tissue of origin for the 24 adult cancer types from TCGA included in the clustering analysis based on epifactor expression. Tissue locations are labeled with TCGA abbreviations. Sex-specific tissue locations are shown in purple for female (left panel) and blue for male (center panel). The full names for each cancer type are provided (right panel). **b** Functional categories for the 720 epifactor genes included in this study (top panel). This list of epifactors genes was obtained from the manually curated Epifactors database generated by Medvedeva et al.^23^. The bottom panel shows the overlap of these epifactor genes with the genetically altered cancer genes cataloged in either the COSMIC^24^ or OncokB^25^ databases. **c** The NMF-based clustering^26,27^ analysis workflow is provided. Raw RNA-seq counts for all of the genes in each patient’s tumor for a specific cancer type were normalized using the DESeq2^67^ R package. The most variable epigenetic genes (var_epi) were selected based on a cancer type-specific standard deviation cutoff. This dimensionally reduced counts matrix (patient x var_epi) was used as an input to the NMF R program^27^. **d** PCA plot showing the two clusters (red and cyan) of the BRCA patient tumors (depicted as dots) as determined by the NMF method. The variances explained by principal components (PC) 1 (*x* axis) and PC2 (*y* axis) are plotted. **e** Heatmap showing the fraction of the top NMF genes for the cancer type in the corresponding column that overlaps with the top NMF genes for the cancer type in the corresponding row. Darker colors indicate a higher fraction of overlap. The rows and columns are hierarchically clustered. **f** Heatmap describing the most frequent top NMF genes (rows, genes ranked in decreasing order of frequency) across the 24 cancer types (columns). **a** and **b** were created using Biorender. Supporting information for this figure can be found in Supplementary Figs. 1 and 2, and Supplementary Data 1–4.

We investigated whether tumors from each of the 24 adult cancer types in the TCGA repository (Fig. 1a and Supplementary Data 1)^16^ would separate into well-defined subgroups based on the expression patterns of 720 epifactor genes from the Epifactors database^23^ (Supplementary Data 2). These epifactors encode proteins involved in the addition, removal, and recognition of DNA methylation and histone marks, and chromatin remodeling (Fig.1b, top panel, Supplementary Data 2). The majority of the epifactor genes (556 out of 720) are not known to be genetically altered in cancer tissues (Fig. 1b, bottom panel and Supplementary Data 2)^24,25^. We clustered the patient tumors from each cancer type using the non-negative matrix factorization (NMF) algorithm based on the epifactor genes with the most variable expression among the patient tumors (Fig.1c and Supplementary Data 1)^26,27^. With NMF clustering, a reduced representation of the gene expression data is generated that delineates a subset of genes that are important for separating the samples into clusters. For each of the 24 cancer types evaluated independently, separating the tumors into two clusters resulted in the best solution based on three measures of cluster validation (Supplementary Data 1, Supplementary Fig. 1, and Supplementary Data 3). The two clusters for each cancer type were characterized by a set of signature top NMF genes with distinct expression patterns for the tumors in the two clusters (Supplementary Data 4). As an example, for breast cancer, two distinct tumor clusters were observed (Fig. 1d and Supplementary Fig. 2a), and the PAM50 breast cancer subtypes^28,29^ were non-randomly distributed between the two BRCA epifactor expression-based clusters (Supplementary Fig. 2b), consistent with a previous study showing different epigenetic characteristics for the PAM50 subtypes^30^.

The number of top NMF genes across the 24 cancer types ranged from 76 genes for LGG to 9 genes for CRC, with a median of 43 genes (Supplementary Fig. 2c). A pan-cancer map based on the expression patterns of the top NMF genes from all tumor types showed that the tumors group largely based on their tissues of origin, and, to some extent, tissue proximity (Supplementary Fig. 2d). For example, KIRC and KIRP, two types of kidney cancer, were found near each other, and LGG and GBM, two types of brain cancer, were also adjacent to each other in this low-dimensional representation.

There was a high overlap among the top NMF genes in the ACC (carcinoma of the adrenal glands that sit atop each kidney), BRCA, LIHC (liver), LUAD (lung), STAD (stomach), LGG (brain), UCEC (uterus), and SARC (soft tissues and bone) cancer types (Fig. 1e). A strong overlap was also observed among the top NMF genes in the KIRC (kidney), KIRP (kidney) and PAAD (pancreas) tumors (Fig. 1e). SATB1 was the gene most frequently represented as a top NMF gene and was a signature gene for 12 cancer types (Fig. 1f). SATB1 mediates chromatin organization by acting as a “landing platform” for chromatin remodeling proteins^31^. When the top NMF genes were assigned to one of 19 protein complex groups^23^, epifactors belonging to histone acetyltransferase (HAT) complexes were significantly enriched (*P* < 0.05) among the top NMF genes for seven cancer types (LIHC, GBM, UCEC, BRCA, LUAD, KIRP, and PAAD).

### Epifactor expression-based clusters have different clinical outcomes for ten adult cancer types

**Fig. 2:**
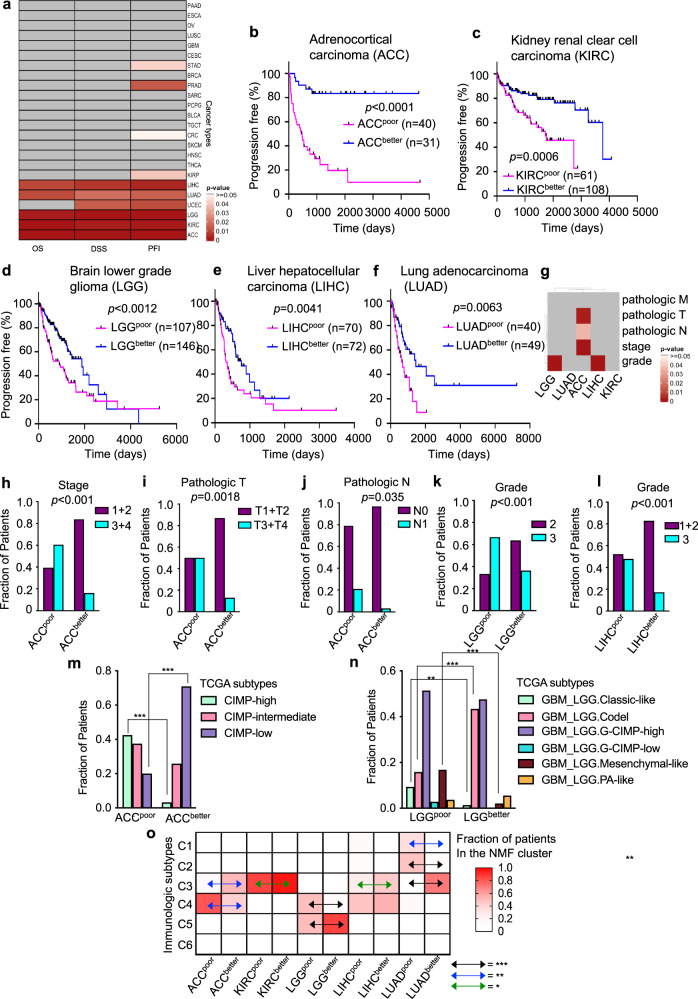
Epifactor expression-based tumor clusters for five TCGA cancer types (ACC, KIRC, LGG, LIHC, and LUAD) correlate strongly with clinical outcome. **a** Heatmap showing the significance (*P* value from multivariate Cox regression analysis; adjusted for age and sex) of the difference in the clinical outcome (PFI, DSS, and overall survival) between the two epifactor expression-based tumor clusters for each of the 24 cancer types. The grey color indicates that the difference in clinical outcome between the two clusters is not significant. **b**–**f** Kaplan–Meier plots comparing the progression-free intervals of the two NMF-derived clusters for the five-cancer group that show significant differences in clinical outcome for the three metrics PFI, DSS, and overall survival. Significance was determined with the log-rank Mantel–Cox test. The cluster with poor outcome is designated (in superscript) “poor,” while the cluster with better outcome is designated “better.” The number of patients (*n*) in each cluster is shown. **b** ACC^poor^ *n* = 40, ACC^better^ *n* = 31. **c** KIRC^poor^ *n* = 61, KIRC^better^ *n* = 108. **d** LGG^poor^ *n* = 107, LGG^better^ *n* = 146, **e** LIHC^poor^ *n* = 70, LIHC^better^ *n* = 72, **f** LUAD^poor^ *n* = 20, LUAD^better^ *n* = 49. **g** Heatmap showing the significance (*P* value; two-tailed Fisher’s exact test) of the difference in clinical metrics (pathologic M, pathologic T, pathologic N, stage, and grade) between the epifactor expression-derived clusters for the five-cancer group. **h**–**l** Barplots of the clinical characteristics for instances (shown in **g**) in which the two clusters significantly differ. **m**, **n** Composition of epifactor expression-derived clusters for ACC (**m**) and LGG (**n**) with regard to established TCGA subtypes. **o** Classification of the epifactor expression-derived clusters for the five-cancer group based on established immunologic subtypes from ref. ^33^ with the following prognostic order (worst to best): C4 ~ C6 > C2 ~ C1 > C3 ~ C5. Data for the clinical metrics were obtained from cBioPortal for Cancer Genomics^60^. Supporting information for this figure can be found in Supplementary Figs. 3–8 and Supplementary Data 5.

To determine whether patients in the two clusters developed from expression levels of epifactors differ with regard to their clinical outcomes, we compared the progression-free interval (PFI), disease-specific survival (DSS), and overall survival of the patients in the two clusters for each cancer type. Cox regression was used to adjust for the effects of age and sex of the patients in each cluster, unless otherwise mentioned. The clusters from 10 out of 24 cancer types (ACC, CRC, KIRC, KIRP, LGG, LIHC, LUAD, PRAD, STAD, and UCEC) had significant differences in clinical outcome (*P* < 0.05) for at least one of the three metrics (PFI, DSS, and overall survival) (Fig. 2a).

For the cancer types in the five-cancer group (ACC, KIRC, LGG, LIHC, and LUAD), the two clusters (Supplementary Figs. 3 and 4) significantly differed in clinical outcome for all three metrics (Fig. 2b–f and Supplementary Fig. 5). Consistent with the differences in outcome, the poor outcome ACC^poor^ cluster was composed of tumors with higher cancer stage (TNM stages 3 and 4), larger size (T3 and T4), and greater likelihood of lymph node spread (N1), compared to tumors in the better outcome ACC^better^ cluster (Fig. 2g–j). The poor outcome LGG^poor^ and LIHC^poor^ tumors also included a significantly higher fraction of patients with grade 3 tumors than the tumors in the LGG^better^ and LIHC^better^ clusters, respectively (Fig. 2g, k, l). When the clinical outcome differences between the NMF clusters were adjusted for stage and grade, all three metrics were still significant, except that for LGG and LIHC, significance was observed for 2 out of 3 metrics (Supplementary Fig 6a). The distributions of the patients’ races and ethnicities did not differ between the clusters (Supplementary Fig 6b–g and Supplementary Data 1) except for LIHC^poor^, which had a higher fraction (~twofold) of Asian patients than LIHC^better^.

The prognostic efficacy of epifactor expression-based clusters was better than grade or epithelial-to-mesenchymal transition (EMT) for the five-cancer group (Supplementary Fig. 7a–d). Tumor grade was effective in predicting the outcome for just 1 or 2 cancer types, out of the five, across the three outcome metrics, while EMT could predict outcome for 2–4 cancer types across the three outcome metrics (Supplementary Fig. 7a–d). The two tumor clusters were not significantly different for EMT for ACC, LGG, LIHC, or LUAD (Supplementary Fig. 7e). For KIRC, we did observe a significant difference (*P* = 0.0015, two-tailed Mann–Whitney test) in the EMT scores between the two clusters, however, the tumors in the poor outcome cluster had a lower EMT score (median = 0.0750) compared to the tumors in the better outcome cluster (median = 0.17), the opposite of the expectation that a more mesenchymal phenotype would be associated with a worse prognosis^32^.

We asked whether the clinical differences between the clusters might reflect differences in the purities of the tumors in the two clusters. There was a significant difference in purity only for ACC and LGG (*P* < 0.05, two-tailed Mann–Whitney test) (Supplementary Fig. 8a), and all three clinical outcomes (PFI, DSS, and overall survival) were still significantly different for both ACC (*P* = 0.002, *P* = 0.006, and *P* = 0.001; Cox regression) and LGG (*P* = 0.008, *P* = 0.007, and *P* = 0.02) after adjusting for tumor purity. The differences in stromal fraction were significantly different for ACC and LGG clusters (Supplementary Fig. 8b), but the clinical outcome differences between the NMF clusters for ACC (*P* = 0.0003, *P* = 0.0006, and *P* = 0.0003; Cox regression) and LGG (*P* = 0.0006, *P* = 0.001, and *P* = 0.003) were still significant after adjusting for stromal fraction. The ACC and KIRC clusters had different levels of immune infiltration (Supplementary Fig. 8c), but the clinical outcome differences between the NMF clusters for ACC (*P* = 0.007, *P* = 0.015, and *P* = 0.002; Cox regression) and KIRC (*P* = 0.003, *P* = 0.005, and *P* = 0.0007) were still significant after adjusting for leukocyte infiltration.

We compared the epifactor expression-derived clusters with established TCGA subtypes for the five-cancer group^33^. None of the clusters were composed of only a single TCGA-defined tumor subtype. But, for each of the clusters, there was at least one TCGA subtype that was overrepresented (Fig. 2m, n, Supplementary Fig. 8d–f and Supplementary Data 5). For example, for ACC and LGG (Fig. 2m, n), there was a greater representation of some DNA methylation-based TCGA subtype(s) in the poor outcome cluster compared with the better outcome cluster, and vice versa. These results indicate that previously reported TCGA subtypes may have epigenetic features that contribute to their distinctive characteristics. Our epifactor expression-derived clusters also contained tumors with significantly different compositions of immunologic subtypes (see “Methods”)^33^ (Fig. 2o and Supplementary Data 5). Epifactor expression-derived clusters with poor clinical outcomes were enriched in immunological subtypes associated with poor prognosis (such as C4) and/or depleted of the subtypes associated with better outcome (such as C3 and C5), consistent with epifactor expression in cancer cells affecting the immune response to the tumor.

We performed a detailed analysis to determine the significant differences (adjusted *P* value < 0.05, Benjamini–Hochberg method) in the frequencies (fraction of affected patient tumors) of mutations and copy number alternations (CNAs) between the two clusters for these five cancer types (Supplementary Data 4). For ACC, there were no significant differences in the mutation or CNA frequencies between the clusters for any gene (epifactor or non-epifactor). For KIRC, the two clusters were different in terms of CNA frequencies for six epifactor and 431 non-epifactor genes. None of these six epifactors (*NPM1*, *UIMC1*, *NSD1*, *HDAC3*, *DND1*, and *TAF7*) were assigned as cluster-defining top NMF genes. No differences in mutational frequencies between the clusters were observed for any gene. For LGG, only three non-epifactor and zero epifactor genes had significant mutational frequency differences, while three epifactor and 58 non-epifactor genes had CNA frequency differences, between the two clusters. The three epifactors (*PRMT8*, *CHD4*, and *ING4*) with CNA frequency differences were not part of the cluster-defining top NMF gene group. For LIHC, none of the genes had a difference in mutational frequencies between the two clusters. Twenty epifactor genes including one top NMF gene (*TONSL*), and 790 non-epifactor genes had significant differences in CNA frequencies between the two clusters. The CNA in *TONSL* affected <16% of patient tumors in both the clusters suggesting that this CNA is unlikely to have a major effect on the observed differences in patient outcome. For LUAD, only *TP53*, an epifactor gene, displayed differences in mutational frequencies between the two clusters (*P* = 0.006; 68% in LUAD^poor^ vs. 21% in LUAD^better^, two-tailed Fisher’s exact test and adjusted for multiple hypothesis correction). None of the genes (epifactor or non-epifactor) showed differences in CNA frequencies between the two clusters. After adjusting for *TP53* mutations, all three clinical outcomes were still significantly different for the two LUAD clusters. These results suggest that the differences in expression levels of signature epifactor genes for the poor and better outcome clusters were unlikely to exclusively reflect mutations or CNAs.

### Top NMF epifactor genes form co-expression networks

**Fig. 3:**
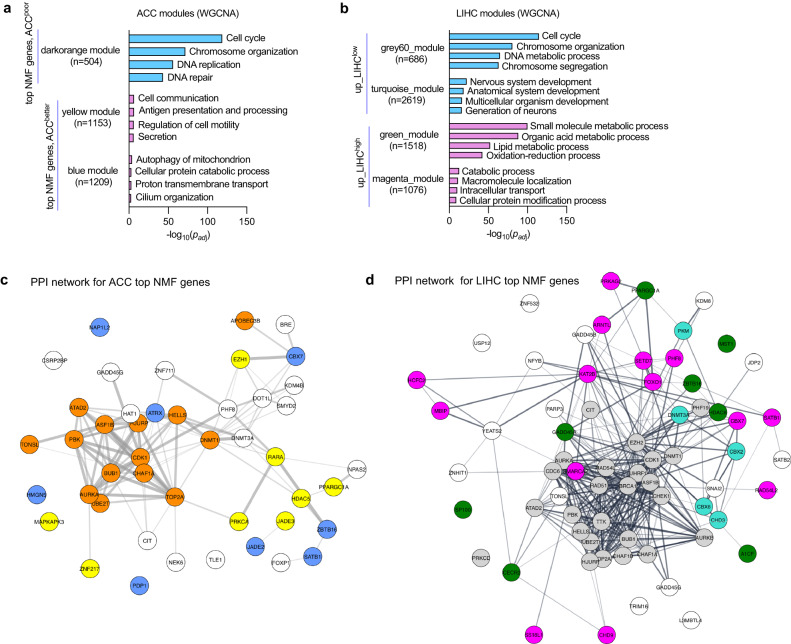
Top NMF gene signatures for clinically-distinct clusters reveal enriched biological functions. **a**, **b** GO terms (bar labels on the right) associated with gene modules containing top NMF genes for ACC (**a**) and LIHC (**b**). Modules were generated using the WGCNA analysis tool^34^ applied to co-expressed genes. Only modules containing at least five top NMF genes were considered. Modules in which top NMF genes are associated with poor outcome are shown in blue, and modules for better outcome are shown in purple. Adjusted *P* values (*P*_adj_) were obtained by applying Benjamini–Hochberg multiple test correction to the unadjusted *P* values in a module. Only the top representative GO terms related to “biological process” or “molecular function” were considered for each gene module. **c**, **d** PPI networks were generated for the encoded proteins of the top NMF genes for the ACC (**c**) and LIHC (**d**) cancer types. The top NMF genes in the networks (nodes; shown as circles) are colored based on the modules in which they reside. Top NMF genes that were not assigned to a module with 5 or more top NMF genes were depicted as white circles (no color fill). The thickness of a line (edge) connecting two top NMF genes (nodes) indicates the confidence level of the protein-protein interaction prediction between those two top NMF genes. Supporting information for this figure can be found in Supplementary Fig. 9 and Supplementary Data 6 and 7.

We performed weighted correlation network analysis (WGCNA)^34^ to identify the gene ontology (GO) terms^35^ associated with gene groups (modules) with similar patterns of expression as the top NMF epifactor genes of poor outcome or better outcome clusters (Fig. 3a, b, Supplementary Fig. 9a–c and Supplementary Data 6). The GO terms for the modules related to poor outcome clusters were enriched for cell cycle genes (dark orange module, ACC; midnight blue module, LGG; grey60 module, LIHC; and green module, LUAD) and developmental genes (turquoise module, LIHC), indicating that differences in proliferation rate or stem-like features^36^ may contribute to the clinical differences observed between the clusters. The protein-protein interaction (PPI) networks formed from the top NMF epifactors were significantly enriched (*P* < 0.05) compared to background for all the five cancer types (Supplementary Data 6, Fig. 3c, d and Supplementary Fig. 9d–f). The top NMF epifactors belonging to the cell cycle-related modules formed tight, well-connected PPI networks indicating a possible coordinated mechanism of action^37^.

### Epifactor expression-based clusters differ in DNA methylation patterns

PCA plots based on array-based DNA methylation levels for the five cancer types^13^ revealed that DNA methylation captures some of the differences between the clusters developed based on epifactor gene expression, but that DNA methylation alone provides significantly less separation between the two tumor clusters than can be achieved by analyzing data from all epifactor genes (Supplementary Fig. 10).

DNA methylation factors tend to be expressed at higher levels in tumors with poor outcome (red color in the heatmap in Supplementary Fig. 11a). This is true for the epifactors that are directly involved in de novo DNA methylation (*DNMT3A* and *DNMT3B*) or in the maintenance of DNA methylation (*DNMT1* and *UHRF1*)^13^. For each tumor type, we determined the number of hypermethylated and hypomethylated loci in the poor outcome cluster compared to the better outcome cluster (Supplementary Fig. 11b and Supplementary Data 7). The pattern of differential methylation between the clusters varied across the five cancer types with more hypermethylation events in the tumors in poor outcome clusters for ACC and LIHC, while the reverse was true for LGG. We determined for all hypermethylated and hypomethylated sites linked to genic regions, whether the closest gene was upregulated or downregulated in the poor outcome cluster (Supplementary Fig. 11c–g). The relationship between DNA methylation state and gene expression levels was significant (Fisher’s exact test) for all cancer types except KIRC, suggesting an impact of DNA methylation levels on downstream gene regulation.

### Expression levels of individual epifactors predict patient outcome

**Fig. 4:**
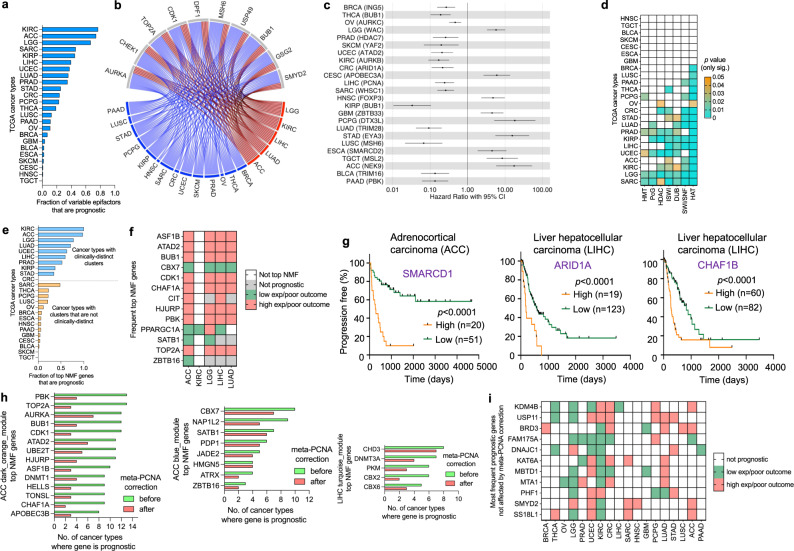
Prognostic potential of epifactor genes depends on the cancer type and in some cancers, is proliferation independent. **a** Number of prognostic epifactor genes for each of the 24 cancer types. Prognostic genes were identified based on a significant difference in PFI outcome between patient tumors with high and low expression levels of the gene. *p* values were adjusted for age and sex of patients and for multiple hypothesis testing (Benjamini–Hochberg method). **b** Circos plot showing the epifactor genes that are most frequently prognostic across cancer types. The lines connect the genes and the cancer types in which they were determined to be prognostic. Red lines indicate the five-cancer group (ACC, KIRC, LGG, LIHC, and LUAD) and blue lines indicate other cancer types. **c** Forest plot showing the hazard ratio and 95% confidence interval (CI) for the most significant prognostic gene (gene symbols in parentheses) for each of the 24 cancer types. Hazard ratios lower than one indicate that higher expression of the gene is associated with poorer PFI. **d** Heatmap for the enrichment of protein complexes among the prognostic genes for the 24 cancer types. White rectangles indicate no significant enrichment. Significance was determined using permutation tests. **e** Barplot showing the fraction of top NMF genes that are prognostic for the 24 cancer types. **f** Heatmap indicating the prognostic status of the most frequent top NMF genes across the five cancer types. **g** Kaplan–Meier plots for the most significant prognostic SWI/SNF genes for ACC and LIHC. Significance was determined with a log-rank Mantel–Cox test and the number of patient tumors (*n*) in each group are provided. ACC^SMARCD1,high^ *n* = 20, ACC^SMARCD1,low^ *n* = 51, LIHC^ARID1A,high^ *n* = 19, LIHC^ARID1A,low^ *n* = 123, LIHC^CHAF1B,high^ *n* = 60, LIHC^CHAF1B,low^ *n* = 82. **h** Bar plots indicating the effect of meta-PCNA correction on the number of cancer types for which a gene is prognostic for the top NMF genes that were included in WGCNA-derived gene modules for ACC (left and center) and LIHC (right). **i** Prognostic status for genes that remain prognostic after the meta-PCNA correction among the cancer types. Supporting information for this figure can be found in Supplementary Data 8.

To complement our clustering analysis based on patterns detected among all of the epifactors, we performed a systematic analysis of the prognostic value of the expression levels of each of the variable epifactor genes (see Methods) considered individually across the 24 cancer types (Supplementary Data 8). The fraction of prognostic epifactors (out of the total number of variable epifactors) varied across the cancer types and ranged from 77% for KIRC to 0.4% for TGCT (Fig. 4a), with a median of 21%. The prognostic direction of a gene was not always the same across the cancer types (Supplementary Data 8) and the expression levels of these prognostic genes among the tumors were not consistently associated with mutations or CNAs that could explain the expression level differences associated with patient outcome (Supplementary Data 8). Among the 24 cancer types, the fractions of prognostic epifactors and non-epifactors were highly correlated (Supplementary Fig. 12a, b), but on average, the fraction of prognostic genes was higher for epifactor genes than for non-epifactor genes (*P* = 0.015, Wilcoxon matched-pairs signed rank test) (Supplementary Fig. 12c). The fraction of variable epifactors that are prognostic among tumor types had a weak negative correlation that did not reach statistical significance (*P* > 0.05) with either the total number of mutations (Supplementary Fig. 13a) or the total number of copy number alterations (CNAs) (Supplementary Fig. 13b).

The top ten most frequent prognostic epifactor genes across the cancer types (Fig. 4b) were involved in chromatin remodeling (*DPF1* and *TOP2A*) and in depositing and reading histone modifications including histone phosphorylation, methylation, and deubiquitination (*AURKA*, *BUB1*, *CDK1*, *CHEK1*, *GSG2*, *MSH6*, *SMYD2*, and *USP49*). Out of these, *AURKA, TOP2A, CDK1*, and *BUB1* were also included in the list of most frequent top NMF genes across the 24 cancer types (Fig. 1f). For the most significant prognostic gene for each of the 24 cancer types, high expression of the prognostic gene was associated with poor outcome for 15 cancer types (hazard ratio <1), while high expression of the prognostic gene was associated with better outcome for nine cancer types (hazard ratio >1) (Fig. 4c). Genes associated with the HAT or chromatin remodeling (SWI/SNF or ISWI) complexes were significantly overrepresented among the prognostic genes (*P* < 0.05) in 11 or more cancer types (Fig. 4d).

For each epifactor, we determined the number of cancer types in which high expression (N) or low expression (M) of that epifactor was associated with poor outcome. Positive prognostic residuals (N-M > 0) were more frequent than negative prognostic residuals, indicating that high expression of epifactors was more often associated with poor outcome for the epifactors overall (“all groups” in Supplementary Fig. 14a, b) and for subsets of epifactors associated with DNA modification (*n* = 25), histone modifications (*n* = 487), and chromatin remodeling (*n* = 124) (Supplementary Fig. 14a, b). We observed a similar trend among epifactors that are histone writers (*n* = 147), erasers (*n* = 57), and readers (*n* = 80) (Supplementary Fig. 14c, d). When we further divided the histone writers based upon the specific histone mark they deposit, the writers that catalyze histone acetylation (*n* = 33), but not those that catalyze methylation (*n* = 47), phosphorylation (*n* = 36), or ubiquitination (*n* = 28), had more negative prognostic residuals than positive residuals, indicating that high expression of histone acetylases is associated with a more favorable prognosis (Supplementary Fig. 14e, f).

A higher fraction of the top NMF genes for the five-cancer group were prognostic for outcome as compared with other cancer types (Fig. 4e, f). Further, the prognostic direction of these frequent top NMF genes was consistent across the five-cancer group, with the exception of *PPARGC1A* (Fig. 4f). The frequent top NMF genes *ASF1B*, *ATAD2*, *BUB1*, *CDK1*, *CHAF1A*, *HJURP*, *PBK*, and *TOP2A* (Fig. 4f) that were signature genes for the poor outcome cluster in ACC, LGG, LIHC, and LUAD (Supplementary Fig. 4 and Supplementary Data 4) were also significantly associated with poor outcome when expressed at high levels for those same cancer types (Fig. 4f). The prognostic genes for ACC and LIHC had a shared enrichment for SWI/SNF chromatin remodeler genes (Fig. 4d) with *SMARCD1* in ACC, and *ARID1A* and *CHAF1B* in LIHC, being the most significantly prognostic SWI/SNF genes (*P* < 0.0001) in these two cancer types (Fig. 4g). The top NMF epifactors were more likely to be predictive of outcome than SWI/SNF epifactors, overall (Supplementary Fig. 15a, b).

In their investigation of genes that predict breast cancer outcome, Venet et al. found that the prognostic value of the majority of signature genes was eliminated when they adjusted for the expression levels of a “meta-PCNA” signature that removed the confounding effects of cell proliferation^38,39^. After adjusting for the meta-PCNA signature, in addition to age and sex, we found that the expression levels of some prognostic epifactor genes were no longer associated with clinical outcome (Supplementary Data 8). Analysis of the instances in which an epifactor gene was “proliferation-independent” (prognostic even after meta-PCNA correction) or “proliferation-dependent” (not prognostic after meta-PCNA correction) revealed that the top NMF genes belonging to a co-expression module (Fig. 3a, b and Supplementary Fig. 9a–c) with a highly enriched “cell cycle” GO term (such as the dark orange module of ACC) were more affected by meta-PCNA correction compared to top NMF genes in modules highly enriched for other GO terms such as autophagy (the blue module of ACC) or development (the turquoise module of LIHC) (Fig. 4h). The frequently prognostic epifactor genes that were also unaffected by the meta-PCNA correction included those involved in histone modifications (*KDM4B*, *KAT6A*, *MBTD1*, *MTA1*, and *PHF1*), histone binding (*BRD3*, *MBTD1*, and *PHF1*), and chromatin organization and remodeling (*MTA1*) (Fig. 4i).

### A machine learning model based on epifactor expression predicts clinical outcome of the adult patients from the five-cancer group

**Fig. 5:**
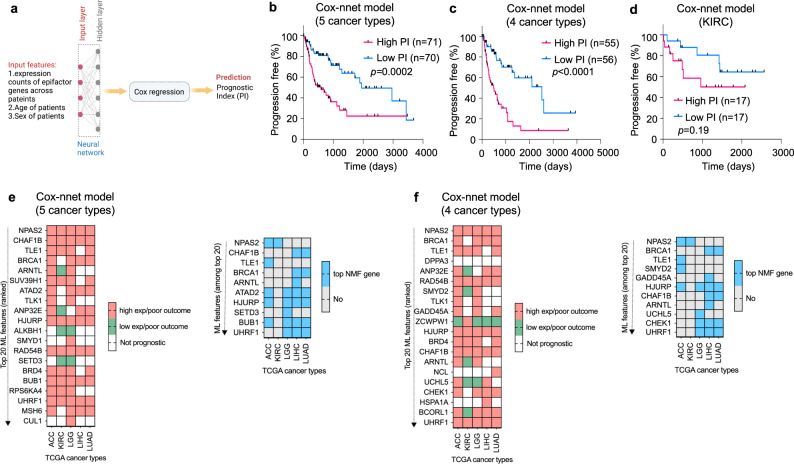
Pan-cancer neural network model predicts patient outcome based on epifactor gene expression patterns. **a** A Cox-nnet model^40^ was used as a framework for predicting patient outcomes. The patient cohort was randomly split (80:20) into training and test sets. The model was trained on input features consisting of the expression values of the 720 epifactor genes, and the age and sex of the patients in the training set. The model consisted of an “input layer” that accepts the input features and is fully connected to a “hidden layer.” The output of the nodes of the “hidden layer” was fed to a “cox-regression layer.” The final output of the model was the log hazard ratios of the patients (prognostic index, PI). To evaluate the performance of the model, the test set patients were divided into high PI and low PI groups based on the median PI of the patients. The clinical outcomes between these two groups were compared using the log-rank Mantel–Cox test (Kaplan–Meier method). Created using Biorender. **b**–**d** Kaplan–Meier plots evaluating the performance of the model. **b** Results when the model was trained and tested on patients from the 5-cancer group (ACC, KIRC, LGG, LIHC, and LUAD). High PI *n* = 71, Low PI *n* = 70. **c** Results when the model was trained and tested on four cancer types (ACC, LGG, LIHC, and LUAD). High PI *n* = 55, Low PI *n* = 56. **d** Results for a model trained and tested on only KIRC. High PI *n* = 17, Low PI *n* = 17. **e** Prognostic status of the top 20 input features (left panel) ranked on the basis of their importance in the Cox-nnet machine learning (ML) model for the five cancer types is shown. A heatmap indicating which of the top 20 features from the left panel are also top NMF genes across the five cancer types is shown on the right. Only the features that are a top NMF gene for at least one cancer type are shown. **f** Same as (**e**), but for the four cancer type model. KIRC was not included in the Cox-nnet model, but is included in these heatmaps for comparison. Supporting information for this figure can be found in Supplementary Data 9.

We asked whether pan-cancer epigenetic features can be used to develop a predictor for patient outcome for the five-cancer group. To achieve this, we used the Cox-nnet artificial neural network (ANN) framework by Ching et al.^40^. The Cox-nnet model consists of an input layer, a hidden layer with 143 nodes, and a final Cox-regression layer that outputs the prognostic index (PI), equivalent to the log hazards ratio (Fig. 5a). Patients from the combined cohort of the five cancer types were randomly split (80:20) into training and test sets. For the model trained on the epifactor expression data, age and sex of the patients in the 5-cancer group, the clinical outcomes (PFI) for the high PI and low PI groups of the test set were significantly different (*P* < 0.0002) (Fig. 5b), indicating that the trained model was able to successfully predict the likely clinical outcome for patients that were not included in the training set. As the top NMF epifactor genes of KIRC showed less overlap with the remaining four cancer types (Figs. 1e and  4f), we also trained a Cox-nnet model based only on the other four cancer types (ACC, LGG, LIHC, and LUAD). With this 4-cancer-type model, the log-rank *P* value for the test set was highly significant (*P* < 0.0001) (Fig. 5c). The model trained only on KIRC did not result in groups with a significant difference in outcome (*P* = 0.19) (Fig. 5d).

Most of the top 20 important features for clinical outcome from the pan-cancer model (Supplementary Data 9) were individually prognostic (*P* < 0.05; Supplementary Data 8) with higher expression of these features associated with poor outcome (Fig. 5e, f, left panels). About half of these important features (10 out of 20 for the 5-cancer model and 11 out of 20 for the 4-cancer model) were top NMF genes in at least one of the 5 cancer types (Fig. 5e, f, right panels).

To further test and validate our findings on the prognostic role of epifactors, we used independent, publicly available datasets for KIRC, LGG, and LUAD (Supplementary Data 9). For each cancer type, we assigned the tumors in this validation cohort to either poor outcome or better outcome groups (Supplementary Fig. 16a–g and Supplementary Data 9) based on the expression pattern of the top NMF epifactor markers that we determined based on the original datasets (Supplementary Fig. 4). In the case of KIRC and LUAD, we observed significant clinical differences (*P* < 0.05; Cox regression, adjusted for age and sex) between the two groups of tumors (Supplementary Fig. 16b–g), while for LGG, the difference was nearly significant (*P* = 0.071) (Supplementary Fig. 16a). There was also a significant overlap (*P* < 0.05, based on the hypergeometric distribution) between the epifactors that were individually prognostic for the validation and primary datasets (Supplementary Fig. 16h and Supplementary Data 9) for KIRC (*P* = 0.019), LGG (*P* = 0.0003), and LUAD (*P* = 0.014). These results demonstrate that expression levels of these epifactors, together or individually, have a robust capacity to classify tumors based on clinical outcomes.

### Epifactor expression-based clustering of pediatric tumors predicts patient outcome

**Fig. 6:**
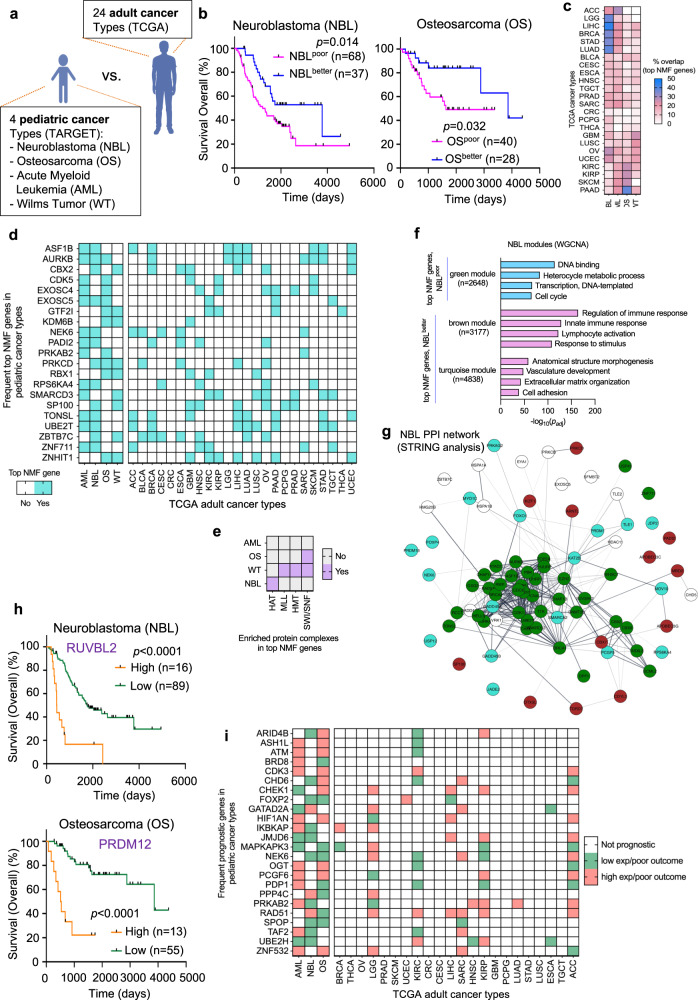
Comparison of pediatric and adult tumors reveals common and distinct epigenetic characteristics. **a** Schematic depicting the four high-risk and hard-to-treat pediatric cancer types (NBL, OS, AML, and WT) from the TARGET program in this study. These cancer types originate in brain (NBL), bone (OS), immature white blood cells (AML), and kidney (WT) in children and adolescents. These pediatric tumors were compared with 24 adult cancer types (depicted in Fig. 1a) from TCGA. **b** Two epigenetic expression-based clusters showed significantly different survival outcomes for NBL and OS (Kaplan–Meier survival plots). NBL^poor^ *n* = 68, NBL^better^ *n* = 37. OS^poor^ *n* = 40, OS^better^ *n* = 28. **c** Heatmap showing the fraction of cluster-defining top NMF genes for four pediatric cancer types (columns) that overlap with the top NMF genes of the 24 adult cancer types (rows). **d** Heatmap showing status of the most frequent pediatric top NMF genes (rows) as top NMF genes for the different pediatric and adult cancer types. **e** Enrichment of different protein complexes in the top NMF genes of the four pediatric cancer types. Only the significantly enriched complexes (purple) are shown. Significance was calculated using the two-tailed Fisher’s exact test. **f** Barplot indicating the GO terms for three different gene modules for NBL obtained from the WGCNA analysis. These modules included at least 11 top NMF genes that were either all upregulated in the poor outcome or better outcome clusters obtained from the NMF algorithm. **g** PPI network generated using the top NMF genes of NBL. The genes are color-coded based on their associated WGCNA modules (shown in **f**). Thicker edges (connecting lines between the genes (nodes)) indicate higher degree of PPI between the two genes connected by the edge. **h** Kaplan–Meier survival plots for the most prognostic gene for NBL (*RUVBL2*) and OS (*PRDM12*). NBL^RUVBL2,high^ *n* = 16, NBL^RUVBL2,low^ *n* = 89. OS^PRDM12,high^ *n* = 13, OS^PRDM12,low^ *n* = 55. **i** Heatmap indicating the prognostic value of the most frequent pediatric prognostic genes (rows) across the pediatric and adult cancer types (columns). White indicates the gene is not prognostic; green indicates that low expression is associated with a poor outcome; pink indicates that high expression is associated with poor outcome. For (**b**, **h**), the *P* values from the log-rank Mantel–Cox and the number of patients in each cluster (*n*) are indicated. Supporting information for this figure can be found in Supplementary Fig. 7 and Supplementary Data 10 and 11.

Mutation frequencies are estimated to be 14 times lower in pediatric than adult cancers^41^. In one detailed genomic study, for 10% of pediatric tumors, no underlying, cancer-promoting mutation or structural copy number variant could be identified^41^. From this perspective, pediatric tumors have the potential to be more epigenetically driven than adult tumors. To compare our findings on epifactors in adult tumors with pediatric cancers, we obtained genomic and clinical data for pediatric tumors from four high-risk cancer types (neuroblastoma (NBL), osteosarcoma (OS), acute myleoid leukemia (AML), and Wilms tumor (WT)) in the Therapeutically Applicable Research to Generate Effective Treatments (TARGET) datasets (Fig. 6a). These four pediatric cancer types are difficult to treat and originate in different tissues and cells within the body: immature nerve cells of the sympathetic nervous system (NBL); bone (OS); immature white blood cells of the bone marrow (AML); and kidney (WT).

NMF clustering based on expression levels of epifactors in the pediatric patient tumors resulted in two clusters for each of the four pediatric cancer types (Supplementary Data 10). The two clusters were significantly different in overall survival (corrected for age and sex, Cox regression) for NBL (*P* = 0.024) and OS (*P* = 0.032) (Fig. 6b and Supplementary Fig. 17a–d), but not for AML (*P* = 0.092) and WT (*P* = 0.693). The top NMF genes for the four pediatric cancer types (81 top NMF genes for NBL; 34 for OS; 27 for AML; and 31 for WT) (Supplementary Data 10) overlapped to different degrees (ranging from 0 to 44%) with the top NMF genes of the 24 adult cancer types from TCGA (Fig. 6c). Out of the 21 genes that were a top NMF gene in at least two pediatric cancer types (Fig. 6d), seven genes (*ASF1B*, *AURKB*, *SMARCD3*, *TONSL*, *UBE2T*, *ZBTB7C*, and *ZNHIT1*) were shared with the 27 most frequent top NMF genes in adult cancers (Fig. 1f). Across the 24 adult cancer types, LIHC and OV had the most overlap of 8 genes between their top NMF genes and the frequent pediatric top NMF genes (Fig. 6d, right heatmap). The top NMF genes of the pediatric cancer types were enriched for genes related to SWI/SNF (WT and OS), HMT (WT), MLL (WT), and HAT (NBL) protein complexes^23^ (Fig. 6e). Similar to our findings for the adult cancer types, the signature top NMF genes for the poor outcome clusters of both NBL and OS (Fig. 6b) correlated in expression with cell cycle genes in the green modules of NBL (Fig. 6f) and OS (Supplementary Fig. 17e). Also similar to our findings in adult tumors, the top NMF genes included in the green module for NBL formed a well-connected PPI interaction network (Fig. 6g).

Out of 720 epifactors, 51 genes were prognostic for overall survival in NBL, 98 genes in OS, and 97 genes in AML (Supplementary Data 11). None of the epifactor genes were prognostic for overall survival in WT. Kaplan–Meier plots for the most significantly prognostic genes for NBL (*RUVBL2*) and OS (*PRDM12*) are shown in Fig. 6h. The prognostic value did not change after the meta-PCNA correction for any of the pediatric prognostic genes (Supplementary Data 11). Twenty-four epifactor genes were prognostic in at least 2 of 3 pediatric cancer types (Fig. 6i, left heatmap). The prognostic value (and direction) of these 24 epifactor genes varied across the adult cancer types (Fig. 6i, right heatmap) with the most overlap observed for ACC, KIRC, and LGG, three members of the 5-cancer group.

### Single-cell RNA-seq analysis of LGG and NBL tumors reveals that the epigenetic gene expression pattern is present in individual cells

**Fig. 7:**
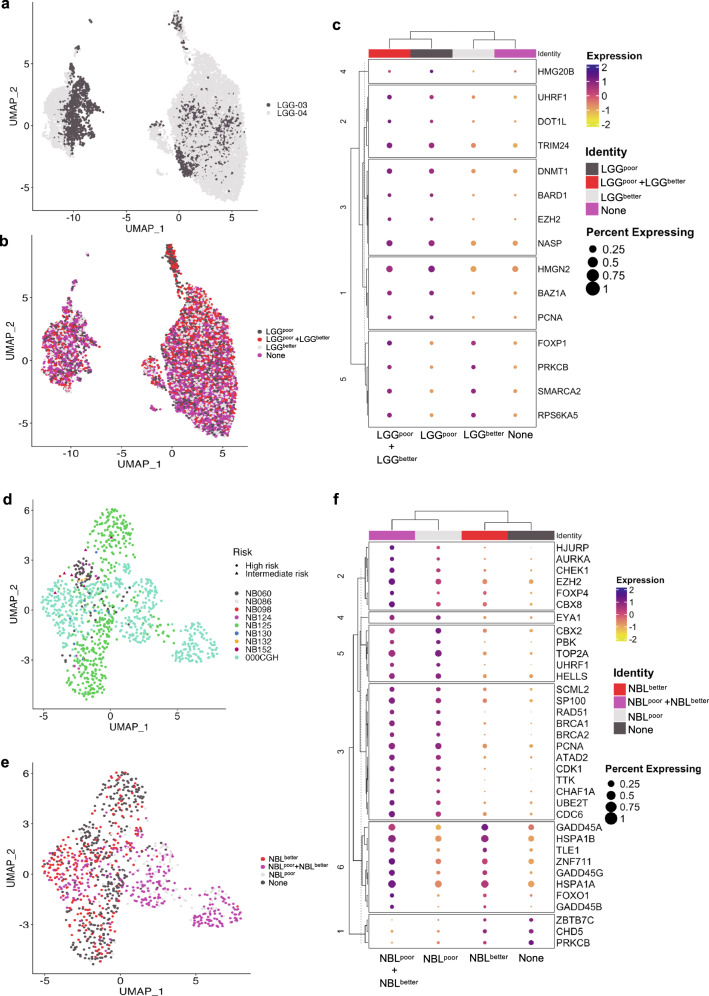
Single cell RNA-seq analysis defines cells expressing epifactor gene signatures of both poor-outcome and better-outcome tumor clusters. **a** UMAP plot of the LGG tumor cells color-coded (grey or black) based on the tumor sample from which they originate (LGG-03 or -04). **b** UMAP plot showing the assignment of tumor cells to four different groups based on the expression levels of the signature genes for the poor outcome (LGG^low^) and better outcome (LGG^high^) tumor clusters for LGG. **c** Dot plot depicting the expression levels and the percent of cells expressing the signature genes for LGG^low^ and LGG^high^ tumor clusters across the four groups determined using single-cell analysis. Only the genes that are differentially expressed across the four different groups are used for the plot. **d**–**f** Same plots as (**a**–**c**), respectively, but for NBL tumor cells.

Given our observation that tumors were associated with one epifactor expression-based cluster or another, we asked whether the individual cells within a tumor would display a gene expression profile related to one of the two clusters. Expression-based composite scores derived from the signature genes for each of the two epifactor expression-based clusters (Supplementary Fig. 3c) were mapped to each cancer cell in a two-patient, single-cell RNA-seq dataset for LGG^42^ (see Methods). Individual cancer cells were assigned to one of the four different groups: LGG^poor^, LGG^better^, LGG^poor^+LGG^better^ (“mixed” group with characteristics of both clusters), and “none” (not scoring high for genes enriched in either cluster) (Fig. 7b). Both of the sequenced LGG tumors contained cells from all four groups in different proportions (Fig. 7a, b). The signature top NMF genes of LGG were differentially expressed in cells in the four groups (Fig. 7c). In a similar analysis of a pediatric single-cell dataset for NBL^43^ (Fig. 7d), individual cells contained patterns of NBL^poor^, NBL^better^, both NBL^poor^ and NBL^better^, and neither (Fig. 7e, f), thus, supporting these epifactors as possible determinants of cellular states.

## Discussion

In this study, we used the pattern of epifactor gene expression as the basis for separating patient tumors for each of 24 adult cancer types into two well-separated clusters. Using just the signature epifactors that drove the clustering (top NMF genes), we created a low-dimensional pan-cancer map where the tumors clustered predominantly by cancer type, and to some extent, by tissue similarity or proximity (Supplementary Fig. 2d). A previously reported map of TCGA tumors based on genome-wide DNA methylation and genome-wide mRNA expression revealed similar groupings based on organ system and tissue-of-origin, including for the brain, kidney and lung^44^. Our findings show that these same relationships among the tumors can be recapitulated when the clusters are generated based on the expression of a limited number of epifactors.

Out of the 24 cancers, the two clusters generated based on epifactor expression were significantly different for PFI for a subset of 10 cancer types: ACC, CRC, KIRC, KIRP, LGG, LIHC, LUAD, PRAD, STAD, and UCEC. For the five-cancer group (ACC, KIRC, LGG, LIHC, and LUAD), the two clusters differed significantly for all three clinical outcomes measured (PFI, DSS and OS) (Fig. 2a). The top NMF genes for the 10 cancer types with PFI differences overlapped to a greater extent than the remaining 14 cancer types (Fig. 1e). There were also more signature epifactor genes that were individually prognostic for these 10 cancer types compared with the remaining cancer types (Fig. 4e). GO terms associated with signature genes in the poor and better outcome clusters for the five-cancer group were different, indicating the involvement of different biological mechanisms for the different tumor types. These findings suggest that for these 10 cancer types, and especially for the five-cancer group, intertumor epigenetic heterogeneity is more clinically relevant than for the other cancer types.

The signature epifactors were predictive for multiple cancers. Using machine learning, we developed a neural network model for the five-cancer group combined that was highly predictive of outcome. The most informative genes for the model showed significant overlap with cluster-defining signature top NMF genes (Fig. 5e).

Pediatric tumors are more likely to have relatively few mutations^20,41,45–48^, and thus are strong candidates for more epigenetically driven tumors. Previous investigations of epifactor gene mutations revealed some overlap between pediatric and adult cancer types, and some mutations that are characteristic of only pediatric or only adult tumors^41^. A pan-cancer analysis of DNA methylation in adult and pediatric tumors showed a wide range in the fraction of CpGs that are hypermethylated or hypomethylated in adult tumors^19^, and hypermethylation and hypomethylation frequencies for pediatric Wilms tumor were within the range of the adult tumors^19^. Among four pediatric tumors analyzed here, we found clinically relevant clusters for NBL and OS (Fig. 6b). While the top NMF genes for the pediatric tumors were more similar to each other than the adult tumors, the top NMF genes for NBL and OS showed the highest degree of overlap with adult cancer types with clinically-distinct clusters (LIHC, LGG, LUAD, and STAD for NBL; and KIRC and KIRP for OS) (Fig. 6c). Following this trend, the common prognostic genes between NBL, OS, and AML, also showed a high degree of overlap with prognostic genes for each other, and some overlap with the prognostic genes for LGG, KIRC, LIHC, KIRP, and ACC (Fig. 6i). Thus, epigenetic similarities between adult and pediatric cancer types can transcend the shared features from tissue similarity or proximity. Single-cell analysis of the brain-related adult cancer LGG and the pediatric tumor NBL showed that some individual cancer cells exhibit gene expression patterns associated with either better or poor outcome clusters. Our data taken together support a model in which a subset of signature epifactors working together can modulate the chromatin barriers of tumor-suppressing and oncogenic processes, leading to different clinical outcomes.

Many epifactors, such as enzymes involved in DNA methylation, histone methylation, and histone acetylation have been suggested as targets for anti-cancer therapy^49–52^. Our extensive and unbiased survey of 720 epifactor genes revealed several novel genes that may represent possible drug targets. In particular, histone acetyltransferases were enriched among prognostic genes and associated with improved patient outcome. This finding would support a possible benefit for histone deacetylase inhibitors that are being approved for cancer treatment^53^. In addition, the SWI/SNF family of chromatin remodelers^54,55^ was also enriched among the prognostic genes across the 24 adult cancer types (Fig. 4d). SWI/SNF factors bind to gene regions, distal enhancers, and CTCF sites^54^, and their role in evicting and sliding nucleosomes has the potential to affect gene expression levels. Our findings thus suggest these two protein families as possible targets for epigenetics-based cancer therapy.

There are several limitations to our study. The list of 720 epifactor genes is likely not an exhaustive list. Our study does not take into account other mechanisms of intertumor epigenetic heterogeneity such as alternative splicing and post-translational modifications. Additional pediatric cancers would allow us to better understand the contribution of epifactors to pediatric tumors from different anatomical sites. The racial category “white” and the ethnic category “not Hispanic/Latino” are overrepresented in the TCGA tumors, and for many tumors, racial and ethnic information is unavailable. As a result, our data do not allow us to conclude whether the findings of this study are applicable to more diverse populations^56,57^. Determining the genomewide impact of the epifactor expression patterns on epigenetic processes of nucleosome positioning or histone modifications will require further studies.

Despite the above limitations, our pan-cancer analysis of epifactor expression revealed that: (i) Individual epifactor genes can have prognostic value even when they are not mutated or undergo copy number alterations; (ii) Tumor clusters with a similar landscape of genetic changes have different clinical behavior that correlates with the expression patterns of epifactors; (iii) The prognostic value of an epifactor can be independent of tumor cell proliferation; (iv) Epifactor expression levels predict outcome for tumors of some sites, but not others; and (v) Among epigenetic changes, our unbiased analysis revealed a particularly important role for histone acetylation and nucleosome positioning as determinants of patient outcome.

In summary, our pan-cancer study illuminates clinically relevant epigenetic differences among tumors, for both adult and pediatric cancer types, based on the variable expression of epifactor genes. Our analyses add to our growing understanding of the clinical differences between cancer types based on their tissue-of-origins and spatial locations in the body, and the epigenetic contributors to patient outcome for tumors of the same site. The results from this pan-cancer study can be used as a foundation for rational drug design targeted at epigenetic regulators.

## Methods

### Data acquisition

Raw RNA sequencing counts for primary patient tumors from 24 adult and four pediatric cancer types included in the TCGA^16^ and TARGET (https://ocg.cancer.gov/programs/target) programs, respectively, were downloaded from the Genomic Data Commons (GDC) portal (https://portal.gdc.cancer.gov/). Clinical outcome data (PFI, DSS, and overall survival) for adult cancer patients were obtained from ref. ^58^, while overall survival data for pediatric cancer patients were acquired from the TARGET and GDC portals. Clinical characteristics for adult patient tumors were obtained from TCGA publications for the corresponding cancer (https://www.cancer.gov/about-nci/organization/ccg/research/structural-genomics/tcga/studied-cancers). For combined analysis of TCGA cancer types, Cox-nnet^40^ and pan-cancer uniform manifold approximation and projection (UMAP) plots^59^, the batch-corrected and normalized RNA expression data were obtained from the Pan-Cancer Atlas (https://gdc.cancer.gov/about-data/publications/pancanatlas). Mutational and CNA data for adult patient tumors were downloaded from The cBioPortal for cancer genomics (cbioportal.org)^60^. The EMT scores of individual tumors for the 24 cancer types were calculated based on the EMT signature genes^61^ using the gene set variation analysis (GSVA) module of GSVA R package^62^. The stromal and leukocyte fractions of the TCGA tumors were obtained from ref. ^33^. Expression microarray (GSE107850) and clinical data for LGG patients for validation studies were obtained from ref. ^63^. For KIRC and LUAD validation studies, we used TCGA patients with low tumor purity that were excluded from the primary analysis.

Data on the assignment of patient tumors to immunologic and TCGA subtypes were downloaded from ref. ^33^. The immunological subtypes (C1-C6) were determined based on five immune signatures: macrophages/monocytes, overall lymphocyte infiltration, TGF-β response, IFN-γ response, and wound healing. Each of the immune subtypes was associated with a different prognosis and followed the order (from worst to best): C4 (lymphocyte-depleted) and C6 (TGF-β dominant) >C2 (IFN-γ dominant) and C1 (wound healing) > C3 (inflammatory) and C5 (immunologically quiet). The assignments of BRCA tumors from TCGA to PAM50 subtypes were obtained from ref. ^29^.

The 720 epifactor genes and related protein complexes were obtained from the Epifactors database (v1.7.3, https://epifactors.autosome.org/description) created by Medvedeva et al.^23^. Unless specified, we used the GRCh38 (hg38) human genome assembly and GENCODE v22 gene annotation (https://gdc.cancer.gov/about-data/gdc-data-processing/gdc-reference-files) for genomic analysis.

### Patient selection

For all 24 adult cancer types, the patient tumors selected had a value of greater than or equal to 70% for any one of the two tumor purity metrics, consensus purity estimate (CPE)^64^ and Clonal Heterogeneity Analysis Tool (CHAT)^65^, except for pancreatic cancer, for which tumors with ABSOLUTE purity^66^ greater than or equal to 33% were included for this study (Supplementary Data 1). Out of 31 TCGA adult cancer types that were initially investigated, we selected 24 cancer types that had the desired number of patient tumors (>70 tumors) that also met the purity criteria described above. These selection criteria were established to ensure a robust clustering of tumors and for the gene expression patterns to be largely reflective of the cancer cells themselves rather than non-cancerous cells in the tumor microenvironment. The raw RNA-seq counts data for the selected patient tumors were gathered using the GDC command line client (https://github.com/NCI-GDC/gdc-client - gdc-data-transfer-tool-gdc-client). Pediatric patient tumors from TARGET were selected using the following criteria: AML, bone marrow and peripheral blood blast counts >50%; NBL, tumor cellularity >75% and tumor necrosis <30%; OS, tumor cellularity >50% and tumor necrosis <50%; and WM, tumor cellularity > 80% and tumor necrosis <20%.

### NMF clustering

Preprocessing for all datasets included normalization and selection of variable epifactor genes. Normalization of the raw RNA-seq gene expression counts of the patient tumors from a cancer type was performed using the “EstimateSizeFactors” function from the DESeq2 R package^67^, followed by log2 transformation. This patient-gene matrix was then filtered to include only those 720 epifactor genes from the Epifactors database (v1.7.3)^23^ for which the standard deviation of normalized and transformed counts across the patient tumors was greater than a specified cutoff. The standard deviation cutoff was selected so that the patient-gene matrix contained about 500–600 epifactor genes. The list of variable epifactors is provided in Supplementary Data 1 for adult cancer types and Supplementary Data 10 for pediatric cancer types.

The preprocessed patient-gene matrix was used as the input for consensus clustering using the NMF algorithm as implemented in the NMF R package^27^. With NMF clustering, a reduced representation of the gene expression data is generated that delineates a subset of genes that are important for separating the samples into clusters. NMF was run under the default parameters unless specified otherwise in our code (see “Code availability”). We grouped the tumors for each of the 24 tumor types into two, three or four clusters (Supplementary Data 1) and used metrics described below to assess the quality of the clusters formed (Supplementary Fig. 1).

### Clustering validation metrics

We used Euclidean distance as the distance metric and determined the optimal number of patient clusters (*n* = 2, 3, or 4) with three different metrics. Silhouette coefficient and cophenetic coefficient were determined by the NMF program (“cluster_metrics” table of Supplementary Data 1). Silhouette coefficients, quantitative metrics of cluster separateness, range from +1 to −1, with a higher value indicating cluster coherency. The cophenetic coefficient measures the cluster stability and higher values indicate better stability. Connectivity was calculated using the cIValid R package for cluster validation (https://cran.r-project.org/web/packages/clValid/vignettes/clValid.pdf)^68^. The connectivity metric measures how well the clusters are connected and a lower value indicates better connection. Based on these three metrics, the optimal number of clusters for each cancer type (adult or pediatric) was determined to be two.

### Identifying top NMF genes

The top contributing genes for each cluster (signature genes or top NMF genes) were obtained using the “extractFeatures” function in the NMF package. This function selects the top NMF genes based on the scoring criteria defined by Kim et al.^69^. To generate heatmaps of signature NMF genes, the patients and top NMF genes were grouped based on NMF cluster membership, and ordered with Euclidean distance-based hierarchical clustering. The UMAP coordinates of the 24 cancer types based on all top NMF genes were calculated using the “umap” function in the “umap” v0.2.8.0 (https://rdrr.io/cran/umap/) R package.

### Clinical analysis

Survival analyes for the NMF clusters based on epifactor expression were performed using the Survival v3.3.1 (https://cran.r-project.org/web/packages/survival/index.html) and Survminer 0.4.4 (https://rpkgs.datanovia.com/survminer/index.html) R packages, and GraphPad Prism v9.4.1 for macOS (GraphPad Software, San Diego, CA). Patients were stratified based on their NMF cluster membership and compared for PFI, DSS, and overall survival for adult cancer types from TCGA, and for only overall survival for pediatric cancer types from TARGET. We implemented multivariate Cox regression based on the Cox proportional hazard model to adjust for age and sex, unless otherwise mentioned, with the “coxph” function from the Survival package. Significance between the two groups was determined by a log-rank Mantel–Cox test. For the five-cancer group, ACC, KIRC, LGG, LIHC, and LUAD, additional clinical measures, including stage, grade, pathologic T, pathologic M, and pathologic N, were used to compare the NMF clusters. Significant clinical differences between two groups were determined using the two-tailed Fisher’s exact test.

For the analysis of prognostic epifactors, the variable epifactors that were previously selected as input to the NMF clustering program were used (see the selection criteria in the Methods section “NMF clustering” above). An epifactor was considered not prognostic for a cancer type if it was initially excluded from the analysis because its expression did not vary sufficiently across the patients for that cancer type and was therefore not a variable epifactor, or if it was included in the analysis but its resulted in two patient cohorts with no significant difference in clinical outcome (*P* < 0.05) after adjusting for age and sex (Cox regression). An epifactor was considered prognostic for a specific cancer type if there was a significant difference in clinical outcome (PFI metric) between the high and low expression patient tumor groups (*P* < 0.05) after correcting for the age and sex of the patients (Cox regression), and also for multiple hypothesis testing (Benjamini–Hochberg method)^70^. For determining the prognostic value of an epifactor gene in each cancer type, the Survminer R package functions “surv_cutpoint” and “surv_categorize” were called to identify the optimal cutoff in expression for each gene, in order to group patient tumors into “high” and “low” expression groups, before running the multivariate cox regression to adjust for age and sex, unless specified. To identify significant prognostic genes, the significance values were corrected for multiple hypothesis testing within each cancer type using the Benjamini–Hochberg method^70^. For comparison with non-epifactors, the prognostic analysis of a random group of 719 non-epifactor genes (Supplementary Data 8) was performed in the same manner as that of the epifactor genes.

For the meta-PCNA analysis, an expression-based prognostic survival analysis was performed that was identical to the previous analysis, except for the inclusion of an additional covariate that adjusted for the expression of proliferation-related genes (or meta-PCNA signature genes) as described in ref. ^38^. This meta-PCNA signature was comprised of the top 1% of genes with expression patterns that correlate most closely with the expression pattern of the *PCNA* gene, a widely used cell proliferation marker, across 36 tissues. The median of the log_2_ normalized expression values of the meta-PCNA genes was calculated for each patient and added as a covariate for multivariate Cox regression. Expression-based grouping of tumors and prognostic significance were derived in the same manner as previously described.

Detailed explanations of the clinical metrics, grade, stage, pathologic T, and pathologic N can be found here: https://www.cancer.gov/about-cancer/diagnosis-staging/staging and https://www.cancer.gov/about-cancer/diagnosis-staging/diagnosis/tumor-grade.

### Mutation and CNA analysis

For each gene, the fraction of tumors with a mutation in that gene was compared between the two NMF clusters. Only those genes for which at least 10% of patient tumors were affected in any one of the two clusters were included for the analysis. The significance of the fraction of tumors with each mutation between the two clusters was determined using a two-tailed Fisher’s exact test. The significance values for all the genes for a cancer type were corrected for multiple hypothesis testing using the Benjamini–Hochberg method^70^. CNA events (amplifications or deletions) were analyzed in the same manner.

### Top NMF and prognostic gene enrichment analysis

To evaluate the enrichment of top NMF genes for epigenetic protein complexes^23^, we calculated the overlap of top NMF genes with genes from each of the 19 protein complexes. The list of genes in each protein complex were obtained from the Epifactors database^23^. For each cancer, we grouped the cancer’s most variable epifactor genes (input genes for the NMF program) into “top NMF” or not. We then calculated the enrichment of multiprotein complex genes in these two gene groups as the odds ratio from a two-tailed Fisher’s Exact test. We used a permutation test of 10,000 iterations to generate a null distribution of enrichment values that we used to calculate significant enrichment in the actual top NMF genes. In each iteration, we permuted the top/not top NMF gene group labels and calculated the odds ratios for containing the multiprotein complex genes. Significance values were corrected for multiple hypothesis testing for each cancer type using the Benjamini–Hochberg method^70^. Enrichment analyses for the prognostic genes were performed by overlapping the multiprotein complex genes with the prognostic epifactor genes in each cancer type. Enrichment tests were performed as described above.

### Validation analysis

We used KIRC and LUAD TCGA low tumor purity cohorts, as well as an external LGG microarray study (Supplementary Data 9), to test the robustness of our findings in the primary dataset. We used KIRC and LUAD patients that did not meet the purity requirement of our original study and normalized the data in the same manner as the high purity cohorts. We also used the preprocessed LGG microarray data (GSE107850) from ref. ^63^ which was already quantile normalized. Array probes from the Illumina DASL beadchip^71^ were mapped to HGNC gene ids using the illuminaHumanv4.db Bioconductor package^72^. We excluded probes that mapped to multiple genes. For genes that are targeted by multiple probes, we included the probe that had the highest expression in the patient.

To test the robustness of our top NMF genes to stratify cancer patients according to clinical outcome, we used GSVA^62^ to calculate enrichment scores for the poor and better outcome top NMF genes (Supplementary Fig. 4) corresponding to the three cancer types in the validation cohorts. Enrichment for these genes in the tumors was determined by the log-transformed normalized gene expression data for each cancer. We classified each patient tumor into the two clinical outcome categories based on the higher enrichment value. We performed cox regression and log-rank test as described above in the clinical outcome survival analysis to compare the overall survival, DSS, and PFI outcome of the two outcome groups.

We also performed the prognostic analysis of individual epifactors in the validation sets using the same workflow as described above. For KIRC and LUAD, we used the same variable epifactors that we previously used for the primary datasets. For LGG, only those variable epifactors that were present in the microarray data (validation cohort) and RNA-seq data (primary cohort) were used.

### WGCNA co-expression analysis

Weighted correlation network analysis (WGCNA) was used to identify modules of genes that are co-expressed with top NMF genes using WGCNA R package^34^. This analysis was performed for ACC, KIRC, LGG, LIHC, and LUAD adult cancer types from TCGA, and NBL and OS pediatric cancer types from TARGET. All parameters were replicated from scripts 1, 2c, and 4 of the Introductory Tutorial I from the WGCNA creators’ website (https://horvath.genetics.ucla.edu/html/CoexpressionNetwork/Rpackages/WGCNA/Tutorials/). These tutorials include data cleaning, construction of co-expression networks, module detection, and pathway enrichment. PPI networks and their significance compared to a background were obtained by running the STRING^73^ application (stringApp; https://cytoscape.org/cytoscape-tutorials/protocols/stringApp/#/) on Cytoscape^74^.

### DNA methylation analysis

We obtained batch-corrected and normalized 450 K methylation array data for TCGA patients from the UCSC Xena Browser (https://xenabrowser.net/datapages). We obtained DNA methylation subtype information from each cancer type’s TCGA publication. We ran principal component analysis on the methylation probes using PCAtools R package^75^ and overlayed the NMF patient grouping and methylation subtype. Then, we conducted a probe-wise differential methylation analysis between the poor outcome and better outcome NMF clusters using the limma R package^76^. We mapped differentially methylated probes to their nearest genes with the probe annotations from UCSC Xena Browser (https://xenabrowser.net/datapages). We excluded instances in which the methylation site was not associated with any gene or with multiple genes.

### Cox-nnet pan-cancer model

The Cox-nnet artificial neural network (ANN) framework^40^, a subcategory of machine learning that loosely mimics the signal processing by neurons, can extract important features and learn non-linear behavior in the data^40^. The inputs to ANN are high-dimensional gene expression data and other information about a patient tumor, and the output is the prognostic index (log hazards ratio) value. Hidden layers between the inputs and the output can capture latent information, and the Cox-nnet program calculates feature importance scores that provide information regarding the most important genes for predicting survival.

Cox-nnet was used to build a predictive pan-cancer model of patient PFI. Preprocessing, training, cross validation, and testing steps were performed in accordance with the tutorial entitled “KIRC example” from the authors’ website (http://traversc.github.io/cox-nnet/docs/examples/). A model was trained on the log_2_ normalized gene expression data for the ACC, KIRC, LGG, LIHC, and LUAD cancers combined, these same five cancer types excluding KIRC, and just KIRC. For each run, an 80/20 train-test split, stratified based on PFI events, was applied for each cancer type using scikit-learn’s “train_test_split” function. The patients assigned to training and test groups were combined across cancer types to yield one training and one test set for the analysis.

The fully connected architecture of Cox-nnet includes an input layer, one hidden layer, and an output layer. The predictor variables used as inputs for the network include the batch-corrected, normalized, and transformed expression count values of 720 epifactor genes for the patient tumors, and age and sex of the patients. Each predictor was fed into the hidden layer, applied a bias term, and activated with the tanh function. The outputs of the hidden layer were fed into the output layer of width 1. The output yielded the predicted log hazard ratio (or Cox regression term) for the input patient. The output was fed to a partial log likelihood cost function with L2 regularization, and all weights and bias terms were updated through backpropagation. A fivefold cross validation was applied using Cox-nnet’s “L2CVProfile” function to optimize model hyperparameters before training on the full dataset with the “trainCoxMlp” function.

After predicting the test patients’ log hazard ratios using the “predictNewData” function, patients were grouped into high prognostic (high PI) and low prognostic (low PI) groups based on a median log hazard ratio threshold. The Survival and Survminer R packages were used to plot the Kaplan–Meier curves based on the two prognostic groups. The importance of each predictor variable was calculated using the “varImportance” function from Cox-nnet which replaces the variable’s original expression value for a patient with the mean expression value of all patients and measures the difference in the partial log-likelihood cost function.

### Single-cell analysis

Single cell RNA-seq data from two LGG patient tumors were obtained from ref. ^42^. Preprocessed data and cell type metadata were hosted by the Broad Institute’s single-cell portal (https://singlecell.broadinstitute.org/single_cell) with accession GSE182109. Data were normalized using the “NormalizeData” function of the Seurat v4.1.0 R package^77^. Two thousand highly variable features (genes) were included using Seurat’s “FindVariableFeatures” function. Data were scaled using Seurat’s “ScaleData” function. Fifty principal components (PCs) were calculated using “RunPCA.” To correct for technical variation between patients, the “RunHarmony” function of Harmony R package v0.1.0^78^ was applied to the PCs using 10 iterations for convergence, yielding 50 batch-corrected PCs. The Seurat’s “FindNeighbors,” “FindClusters,” and “RunUMAP” functions were used to cluster the cells and plot a UMAP. These functions were run on the PCs before and after batch correction to account for technical variation.

Sixteen single-cell NBL patients were gathered from Kildisiute et al.^43^. Preprocessed data were hosted by the Neuroblastoma Cell Atlas https://www.neuroblastomacellatlas.org/. The dataset was normalized, scaled, clustered, and mapped to cell types. Harmony was run on the PCs using 10 iterations and yielding 50 batch-corrected PCs that were corrected for sample-specific batch effects. Seurat’s “FindNeighbors,” “FindClusters,” and “RunUMAP” functions were applied to cluster and plot the cells. Cells classified as tumor cells were selected to focus on heterogeneity among cancer cells. Cancer cells were clustered and reduced to two UMAP dimensions. To quantify top NMF gene expression, the modular expression of signature top NMF genes for each of the two NMF clusters (poor and better outcome clusters) were measured using Seurat’s “AddModuleScore” function, which calculates the mean expression of a gene group of interest and subtracts it from the mean expression of a randomly defined control set of genes. For each cell, we determined the module expression score for both NMF clusters. A mean module expression cutoff was used to assign cells into one of four classifications: “None,” “poor outcome cluster,” “better outcome cluster,” or “poor outcome cluster+better outcome cluster.” Differential expression analysis was then performed with Seurat’s “FindMarkers” function for each gene, using a two-sided Wilcoxon rank-sum nonparametric test. The two conditions being tested were based on the one-vs-all approach, in which one classification group is compared against the rest of the classifications. Significance in differential expression was measured using a Bonferroni–corrected adjusted *P* value of *P* < 0.05, which is default for Seurat. Differentially expressed top NMF genes were plotted and clustered using Euclidean distance-based hierarchical clustering. The plots for single-cell analysis were created using the scCustomize package (https://samuel-marsh.github.io/scCustomize/)^79^.

### Statistics and reproducibility

Statistical tests used in each analysis are documented in the text, figure legends, and respective method sections. All tests are two-sided with significance level at 0.05. Significant *P* values were adjusted for multiple hypothesis testing when applicable; the FDR significance level was set at 0.05. Statistical analyses were performed using the R programming language and GraphPad Prism. For all box plots, 25th and 75th percentiles were used as the limits, and whiskers extended to the minimum and maximum values. The number of tumor samples used for analyses depended on the availability of publicly accessible datasets. Tumors were selected for inclusion based on tumor purity scores. For RNA-seq and differential methylation analyses, each group contained at least 30 tumors. Key findings were validated in additional datasets that were not part of the primary analysis. The sources of all datasets are provided. Links are provided for software used.

### Reporting summary

Further information on research design is available in the Nature Portfolio Reporting Summary linked to this article.

### Supplementary information

Supplementary Information

Description of Additional Supplementary Files

Supplementary Data 1

Supplementary Data 2

Supplementary Data 3

Supplementary Data 4

Supplementary Data 5

Supplementary Data 6

Supplementary Data 7

Supplementary Data 8

Supplementary Data 9

Supplementary Data 10

Supplementary Data 11

Supplementary Data 12

Supplementary Data 13

Reporting Summary

## Data Availability

The results shown here are based on data generated by the TCGA Research Network: (https://www.cancer.gov/tcga), and the Therapeutically Applicable Research to Generate Effective Treatments (https://ocg.cancer.gov/programs/target) initiative, phs000218. The data used for this analysis are available at https://portal.gdc.cancer.gov/projects. Data used for figures are provided in Supplementary Data 12. Any remaining information can be obtained from the corresponding author upon reasonable request.

## Code availability

All code was written in R, Python, or Bash. Scripts, required software packages, and instructions are available on the Github repository^80^ (https://github.com/collerlab-github/epifactors_cancer) and Supplementary Data 13.
