# Supplementary material for: Pan-cancer landscape of epigenetic factor expression predicts tumor outcome
Source: Commun Biol. 2023 Nov 16;6:1138. doi: 10.1038/s42003-023-05459-w (PMC10654613; doi:10.1038/s42003-023-05459-w)
Supplement: Supplementary file 1 — Supplementary Information [file 42003_2023_5459_MOESM1_ESM.pdf]

Supplementary Information

Pan-cancer landscape of epigenetic factor expression predicts tumor outcome

Michael W. Cheng, Mithun Mitra, Hilary A. Coller

Supplementary Figures

Supplementary Fig. 1

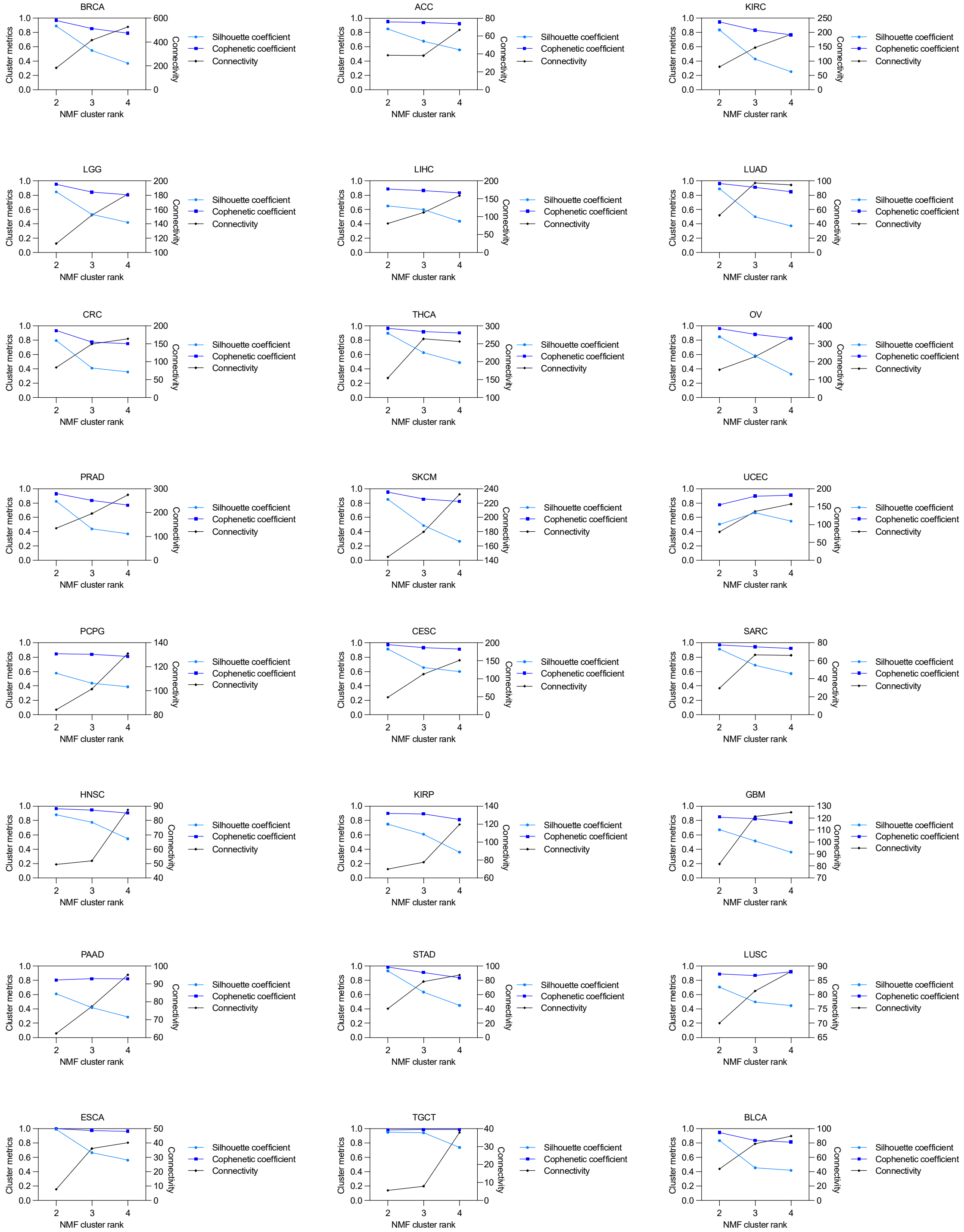

Supplementary Fig. 1 Clustering metrics associated with different numbers of NMF clusters for the 24 cancer types investigated. Related to Fig. 1. For each cancer type, the robustness of the clustering developed for three different numbers (or ranks) of NMF clusters are compared using three different clustering metrics (silhouette coefficient, cophenetic coefficient, and connectivity). A higher value for the silhouette coefficient and the copheneitc coefficient indicates better separated clusters, while a lower value for the connectivity metric reflects better clustering.

Supplementary Fig. 2

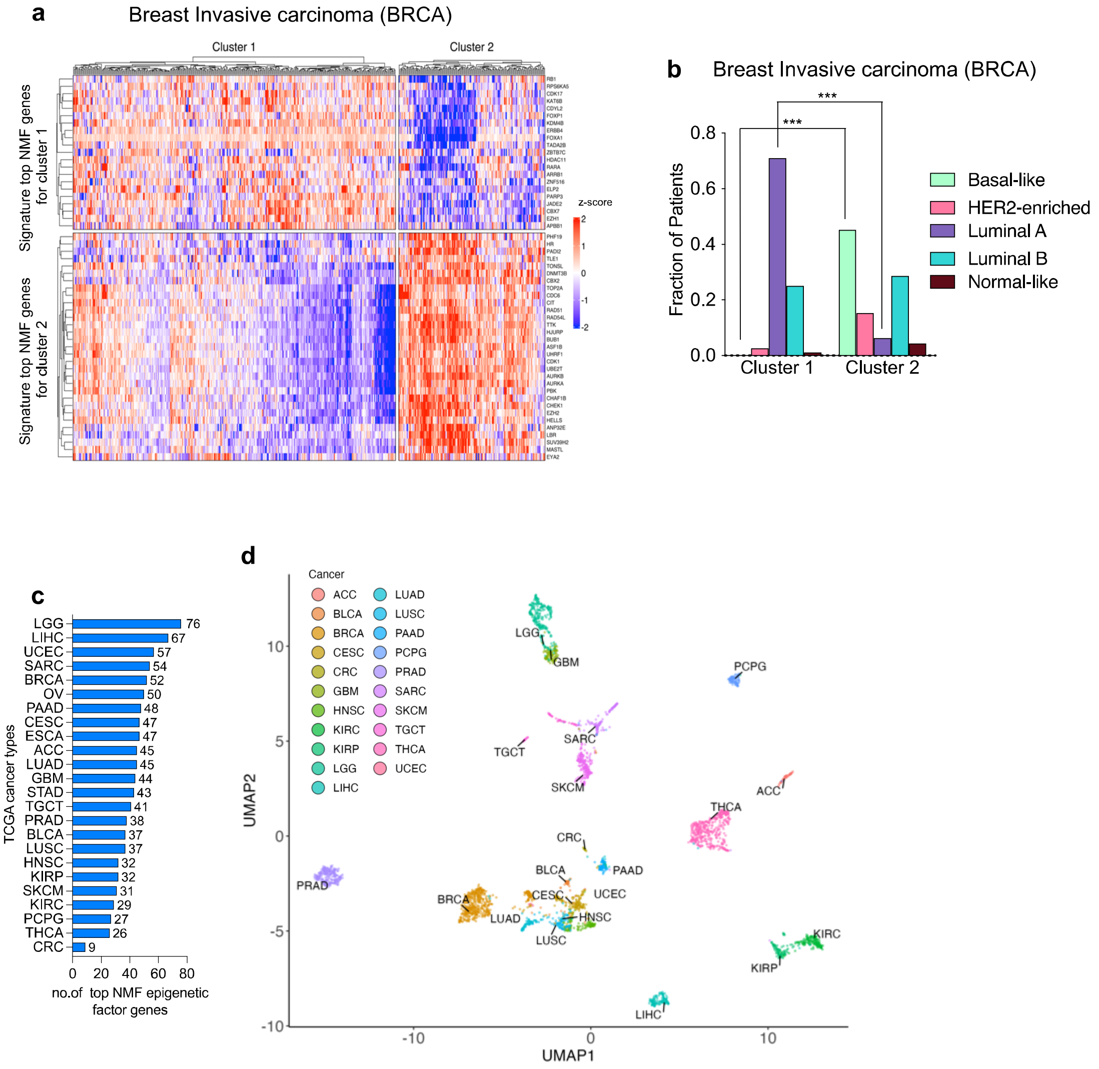

Supplementary Fig. 2 Clustering of patient tumors based on epigenetic factor gene expression and the functions of the genes that contribute to the clustering. Related to Fig. 1. **a** Heatmap showing the expression pattern of the top NMF genes across the two epigenetic factor expression–derived clusters for BRCA. The expression values (log_2(normalized counts)) were scaled (z-scored) before they were plotted. The top NMF genes (rows) were separated into two signature groups that distinguish the tumors in the two clusters. The top NMF genes in each signature group and the tumors in each cluster were hierarchically clustered. **b** Composition of the epigenetic factor expression clusters with regard to PAM50 breast cancer subtypes. **c** Barplot showing the number of top NMF genes for each of the 24 cancer types. **d** UMAP plot showing a low dimensional representation of the patient tumors from 21 TCGA cancer types based on the expression patterns of the top NMF genes for all cancer types.

Supplementary Fig. 3

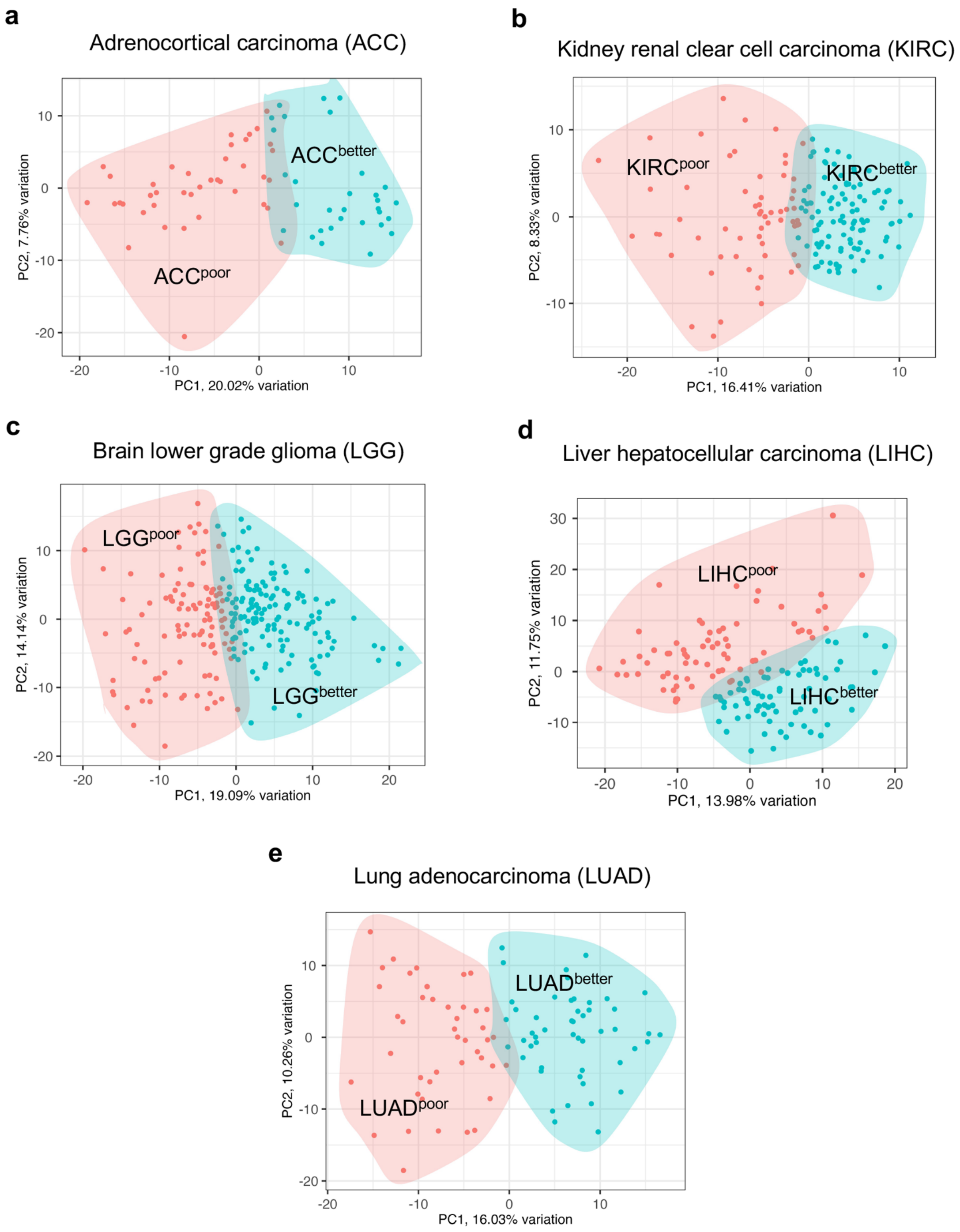

Supplementary Fig. 3 Low-dimensional representation of NMF clusters for tumors with significant differences in outcome. Related to Fig. 2. PCA plots showing the separation of two NMF epigenetic factor expression–derived clusters for ACC (**a**), KIRC (**b**), LGG (**c**), LIHC (**d**), and LUAD (**e**). The patient clusters denoted as poor (red) and better (cyan) are associated with worse and better outcomes, respectively. The percent variance explained by PC1 (x-axis) and PC2 (y-axis) are shown.

Supplementary Fig. 4

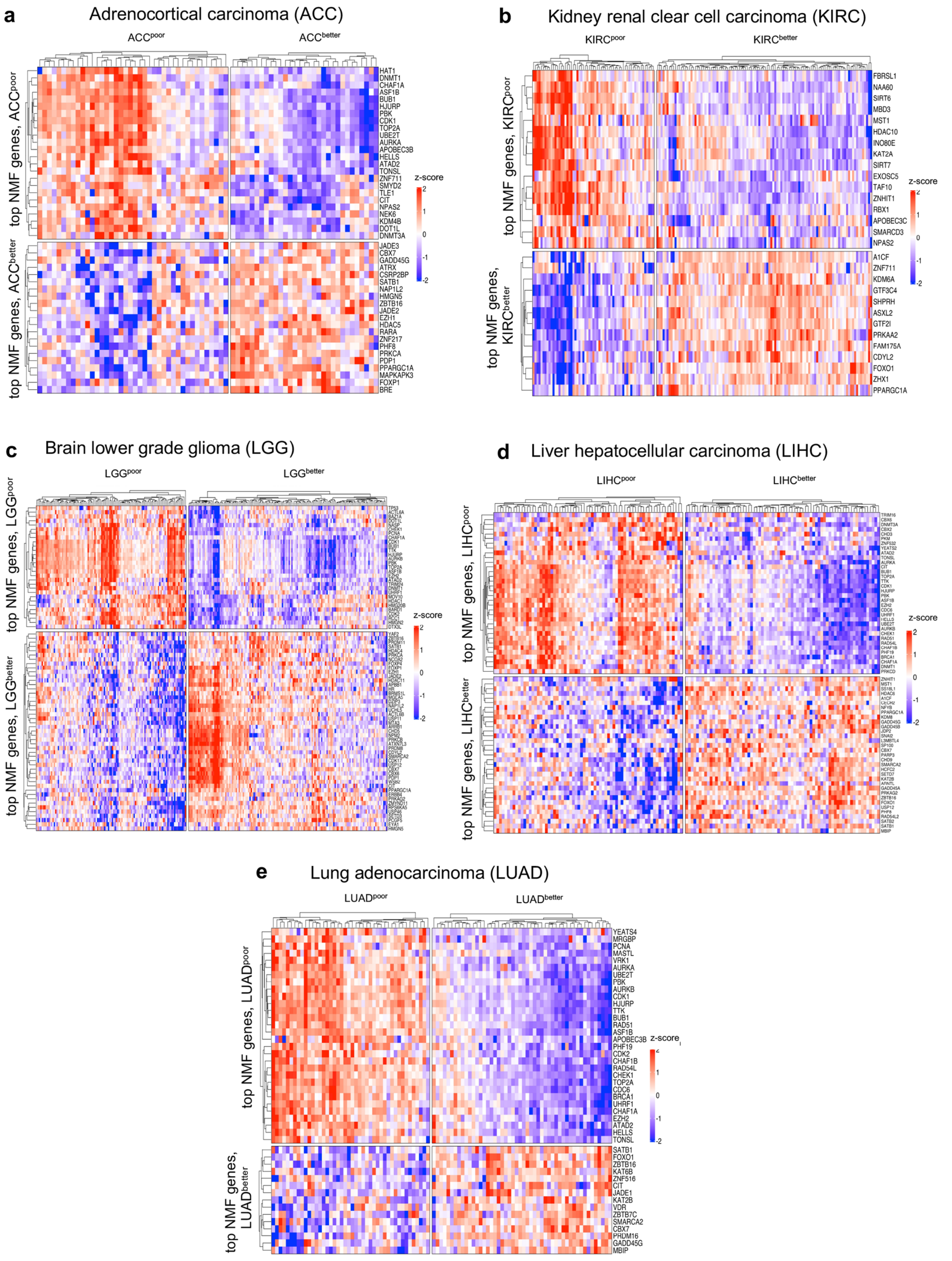

Supplementary Fig. 4 Heatmaps depicting differential expression patterns of the top NMF genes across the two epifactor expression-derived clusters for tumors with significant differences in outcome. **Related to Fig. 2.** Heatmaps showing the expression of top NMF genes across the two NMF epifactor expression–derived clusters. The expression values (log_2(normalized counts)) were scaled (z-scored) before they were plotted. The top NMF genes (rows) were separated into two signature groups that distinguished the tumors in the two clusters. The genes in each gene set and the tumors in each cluster were hierarchically clustered.

Supplementary Fig. 5

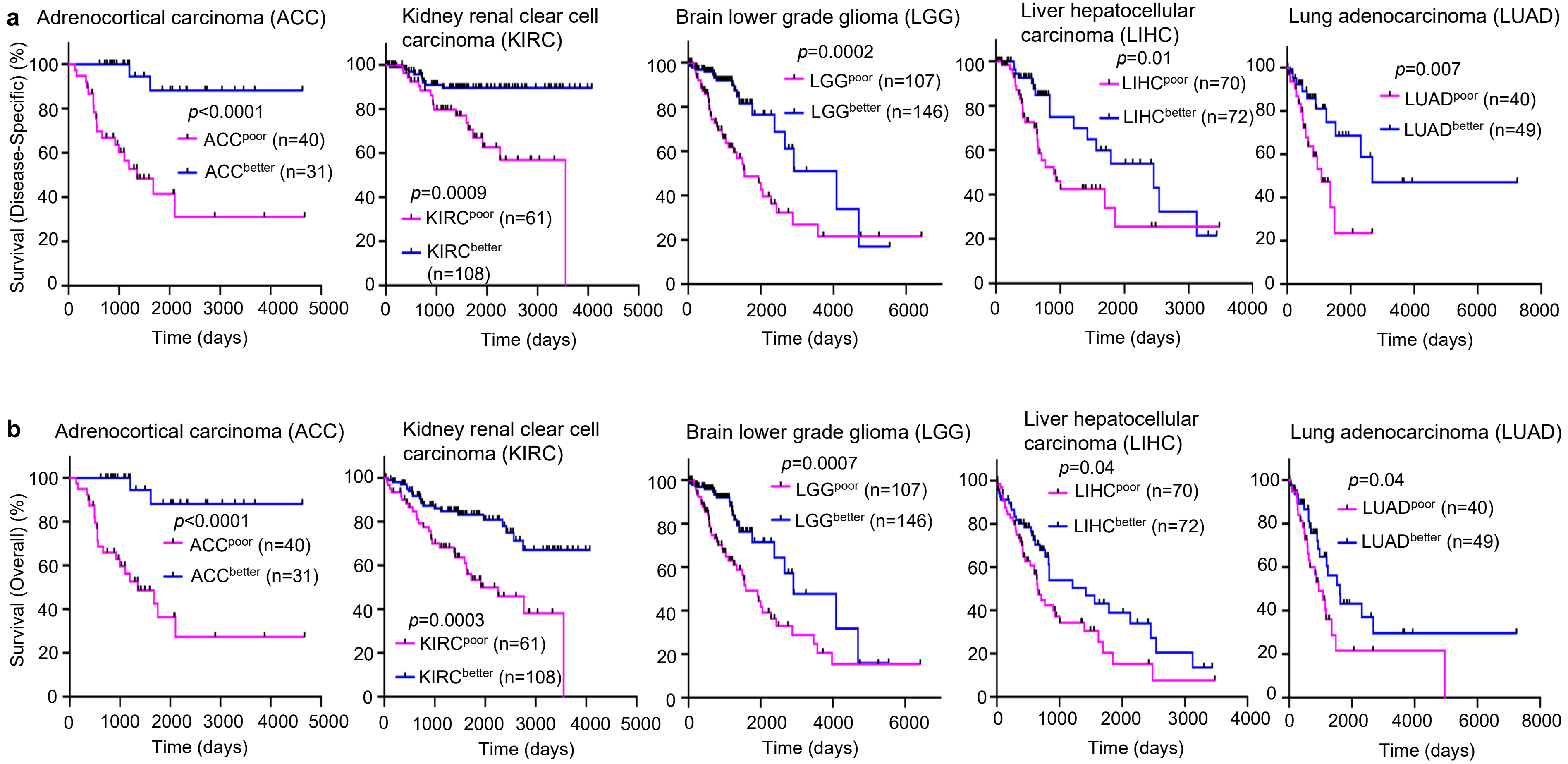

Supplementary Fig. 5 Clinical outcome data (DSS and overall survival) for patients in the two epifactor expression clusters with significant differences in outcome. Related to Fig. 2. Kaplan-Meier plots showing a comparison of the DSS (**a**) and overall survival (**b**) outcome metrics for the two epigenetic factor expression–derived clusters for ACC, KIRC, LGG, LIHC, and LUAD. *p* values were obtained from the log-rank Mantel-Cox test. The number of patients (n) included in each cluster is shown.

Supplementary Fig. 6

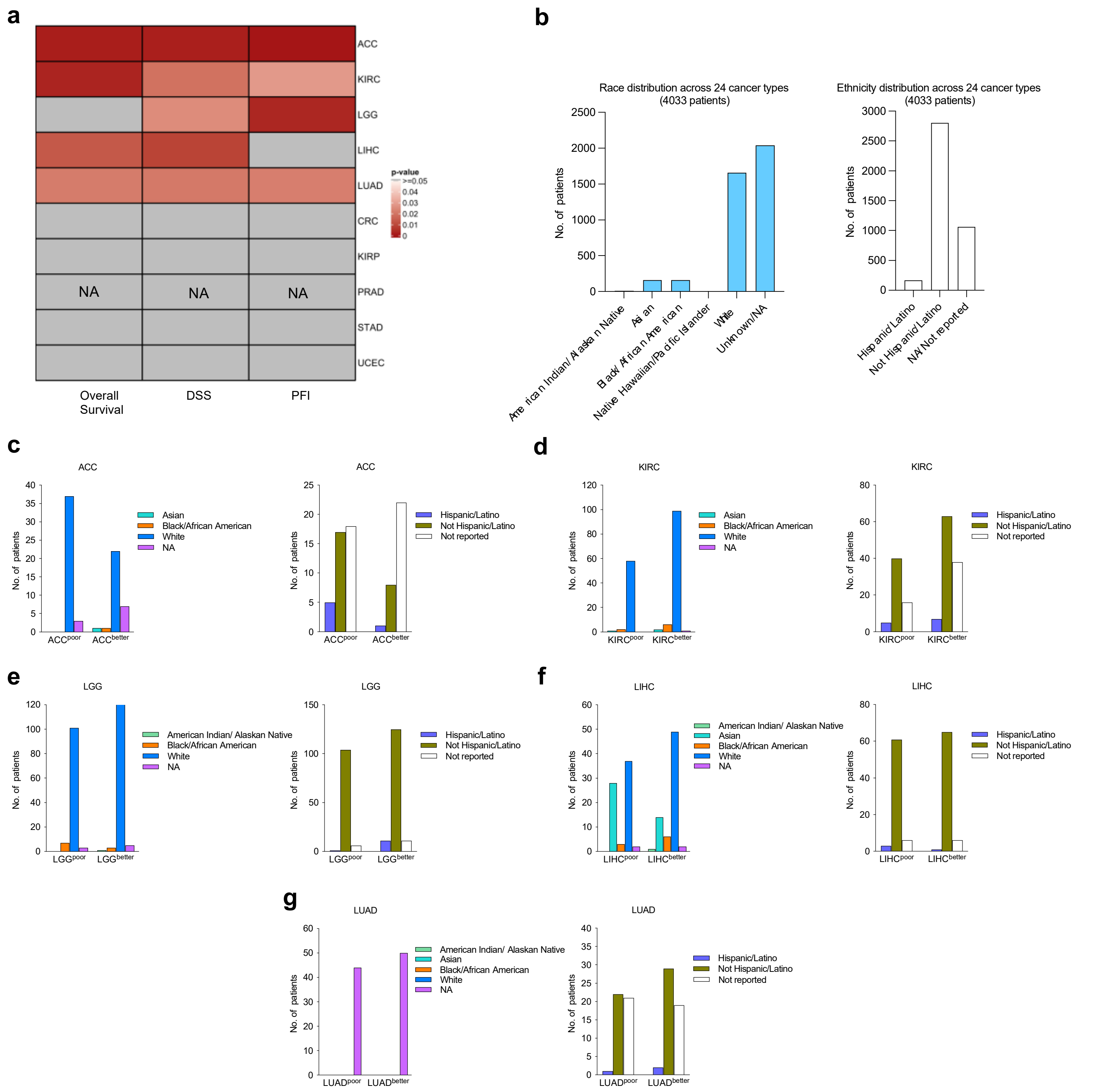

Supplementary Fig. 6 Clinical and ethnicity/race differences between the NMF clusters. Related to Fig. 2. **a** Heatmap showing the significance *p* value for the clinical outcome (overall survival, DSS, or PFI) differences between the two NMF clusters for the ten cancer types. The *p* values (from multivariate Cox regression analysis) were adjusted for grade and stage, in addition to age and sex. The grey color indicates that the difference in clinical outcome between the two clusters is not significant. **b** Race (left) and ethnicity (right) distribution for the combined patient cohort from 24 cancer types. (**c-g**) Comparison of the race (left) and ethnicity (right) distribution between the poor and better clusters based on epifactor expression for ACC (**c**), KIRC (**d**), LGG (**e**), LIHC (**f**), and LUAD (**g**). No information is available for patients in the groups designated as "NA" or "Not reported".

Supplementary Fig. 7

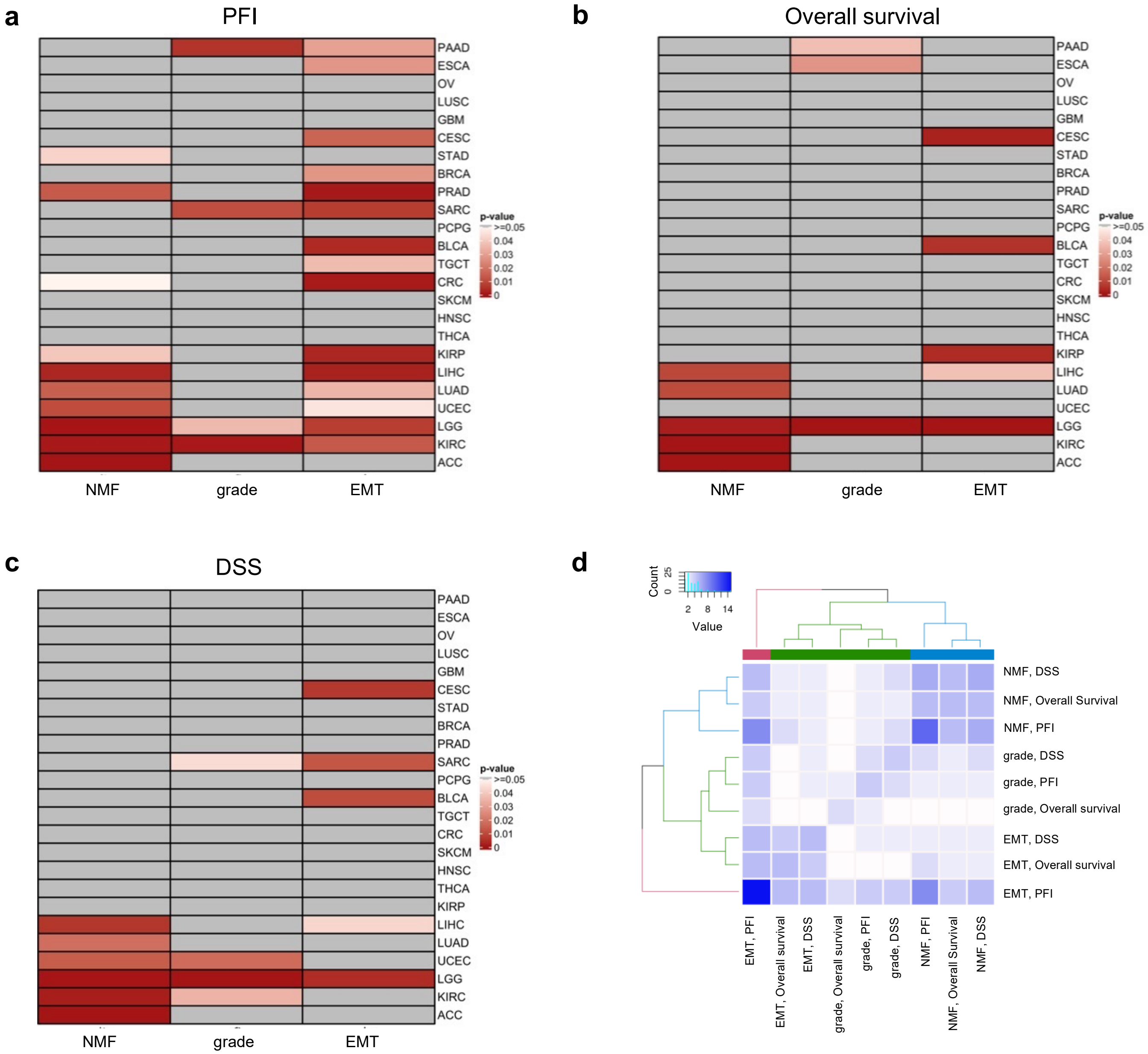

Supplementary Fig. 7 (continued)

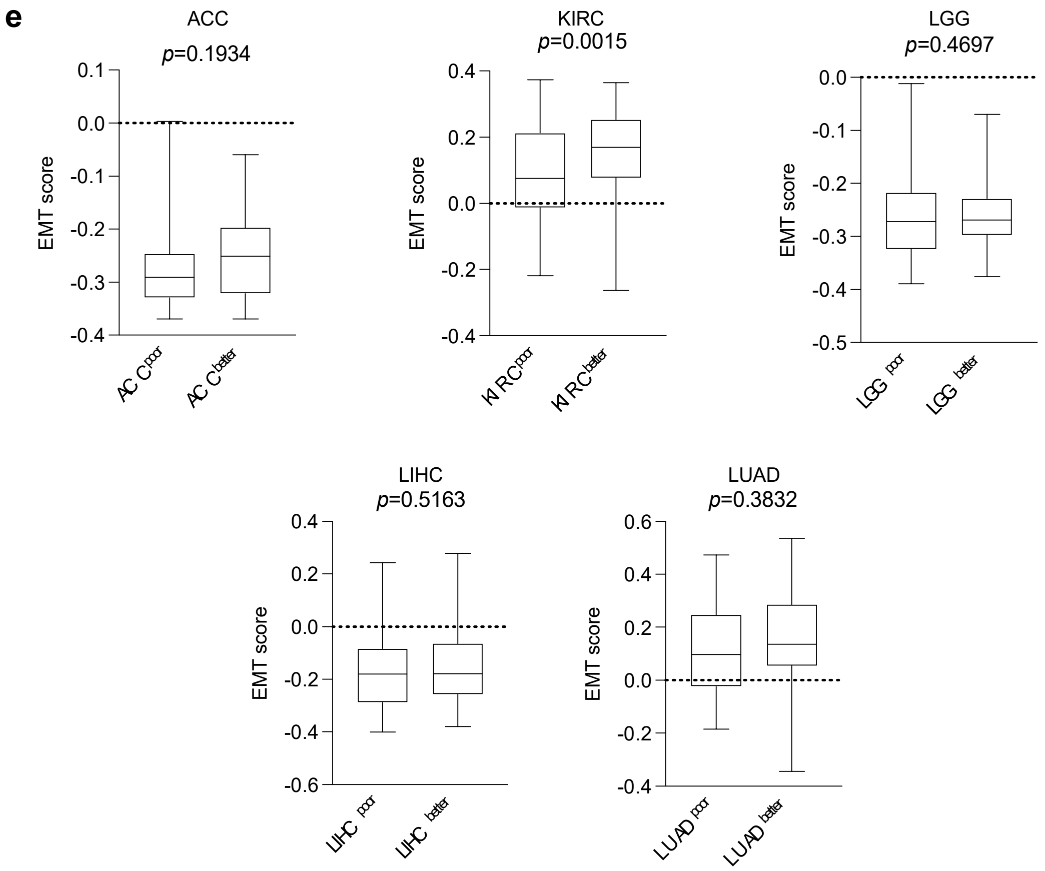

Supplementary Fig. 7 Prognostic efficacy of grade, EMT or NMF clusters for the 24 cancer types. **Related to Fig. 2.** **a-c** Heatmaps comparing significant *p* values for grade, EMT, or epifactor expression–based NMF clusters for the 24 cancer types. The prognostic efficacy is based on PFI (**a**), overall survival (**b**), or DSS (**c**) clinical outcome metrics. The *p* values (from multivariate Cox regression analysis) are adjusted for age and sex. The grey color indicates that the difference in clinical outcome between the two clusters is not significant. **d** Pairwise intersection heatmap of the number of common predictive cancer types depending upon the stratification method (NMF, EMT, or grade) and clinical outcome metric (PFI, overall survival, and DSS) across the 24 cancer types. The rows and columns are hierarchically clustered. **e** Comparison of the EMT scores between the poor and better outcome clusters for the five-cancer group with significant differences in outcome. The *p* values were calculated using two-tailed Mann-Whitney test. The whiskers of the box plots indicate minimum and maximum values.

Supplementary Fig. 8

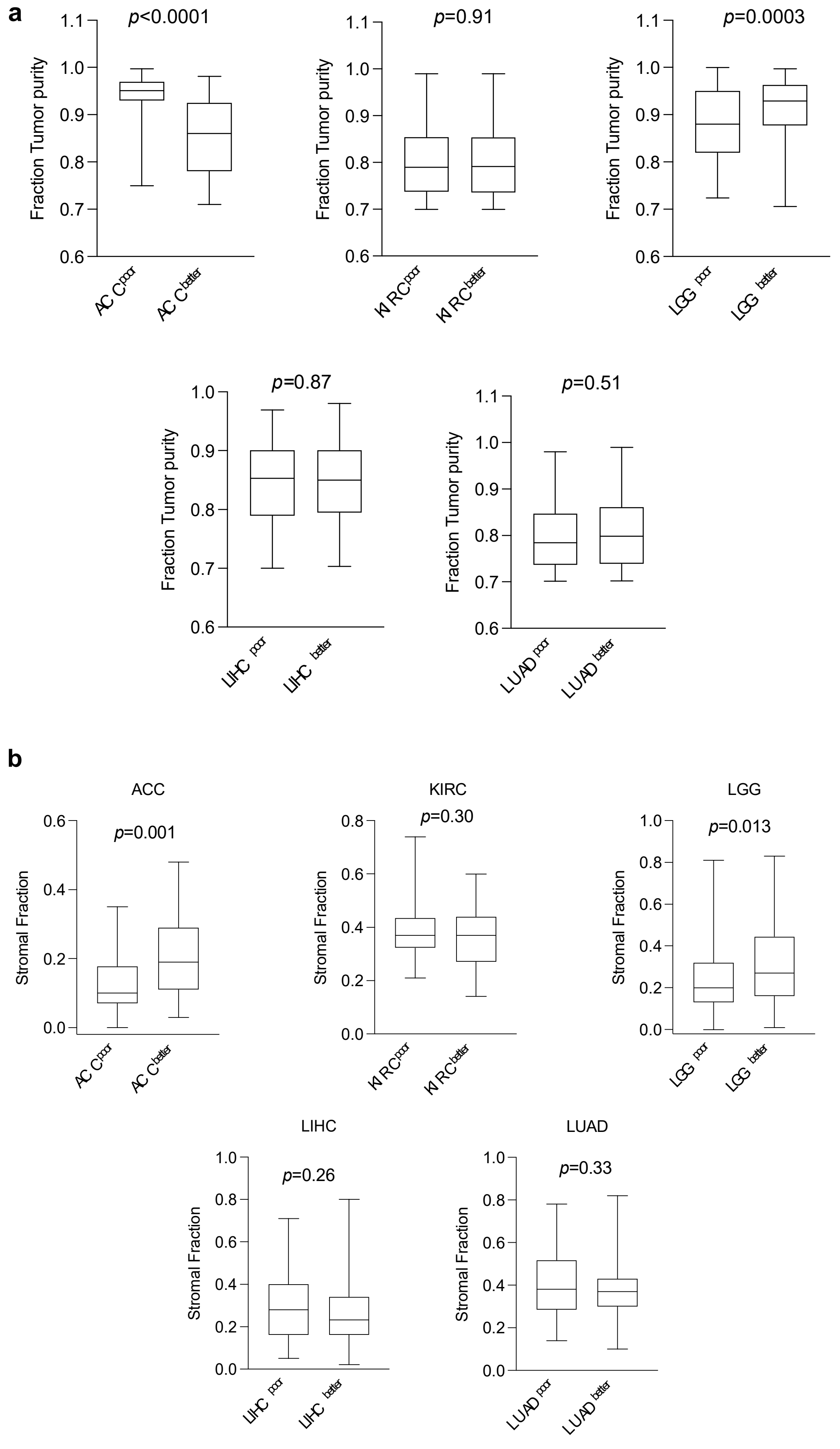

Supplementary Fig. 8 (continued)

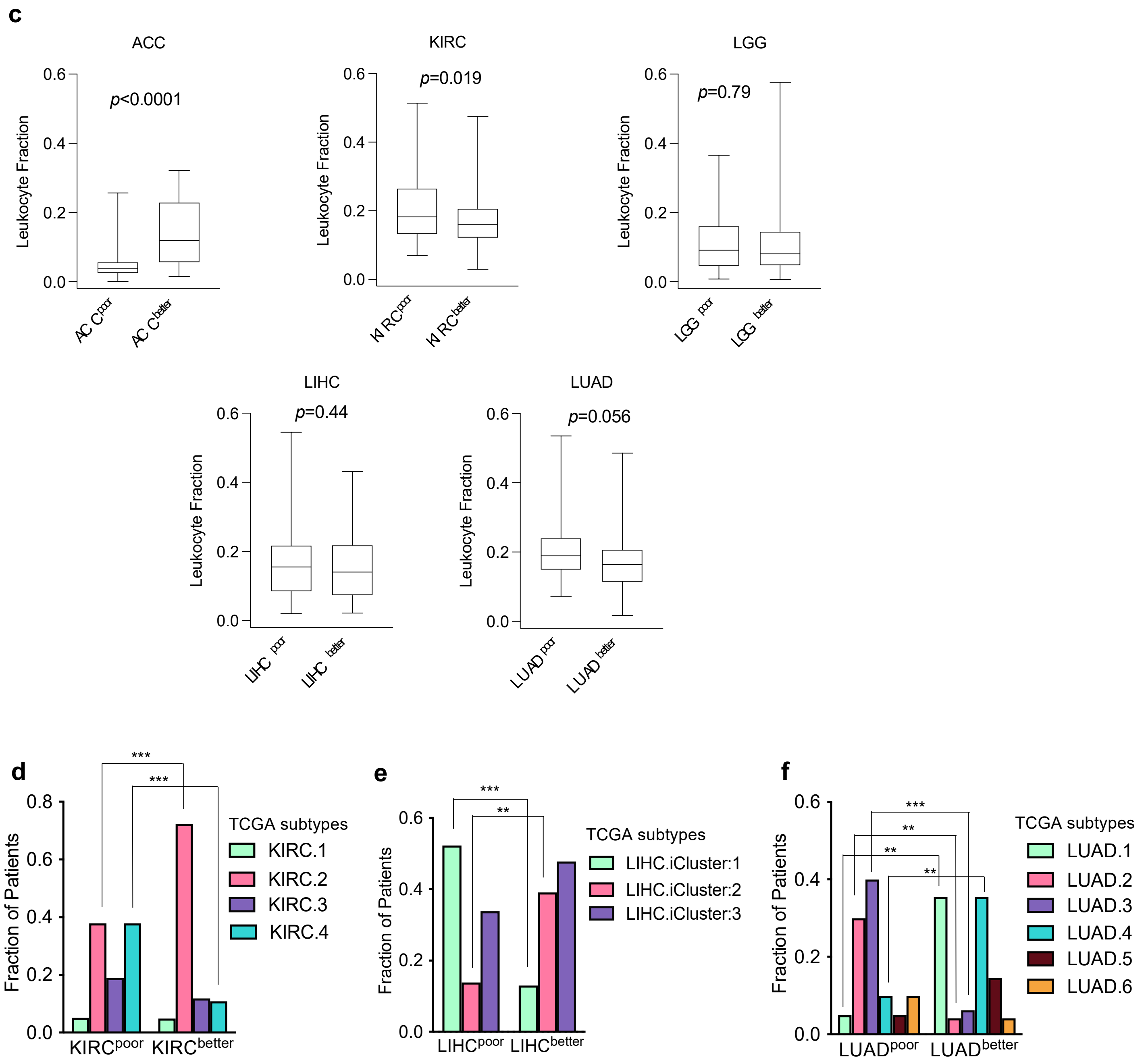

Supplementary Fig. 8 Relationship between the epifactor expression–based clusters with tumor purity, stromal fraction, leukocyte fraction, and known TCGA subtypes. Related to Fig. 2. **a-c** Box and whiskers plots comparing tumor purities (**a**), stromal fractions (**b**), and leukocyte fractions (**c**) between the two epifactor expression-based clusters for the five-cancer group with significant differences in outcome. The p values were calculated using two-tailed Mann-Whitney test. The whiskers of the box plots indicate minimum and maximum values. **d-f** Composition of the two epifactor expression–based clusters with regard to established TCGA subtypes for KIRC (**d**), LIHC (**e**), and LUAD (**f**). Asterisks indicate the significance values (*$p < 0.05$; **$p < 0.01$; and ***$p < 0.001$) (two-tailed Fisher's exact test).

Supplementary Fig. 9

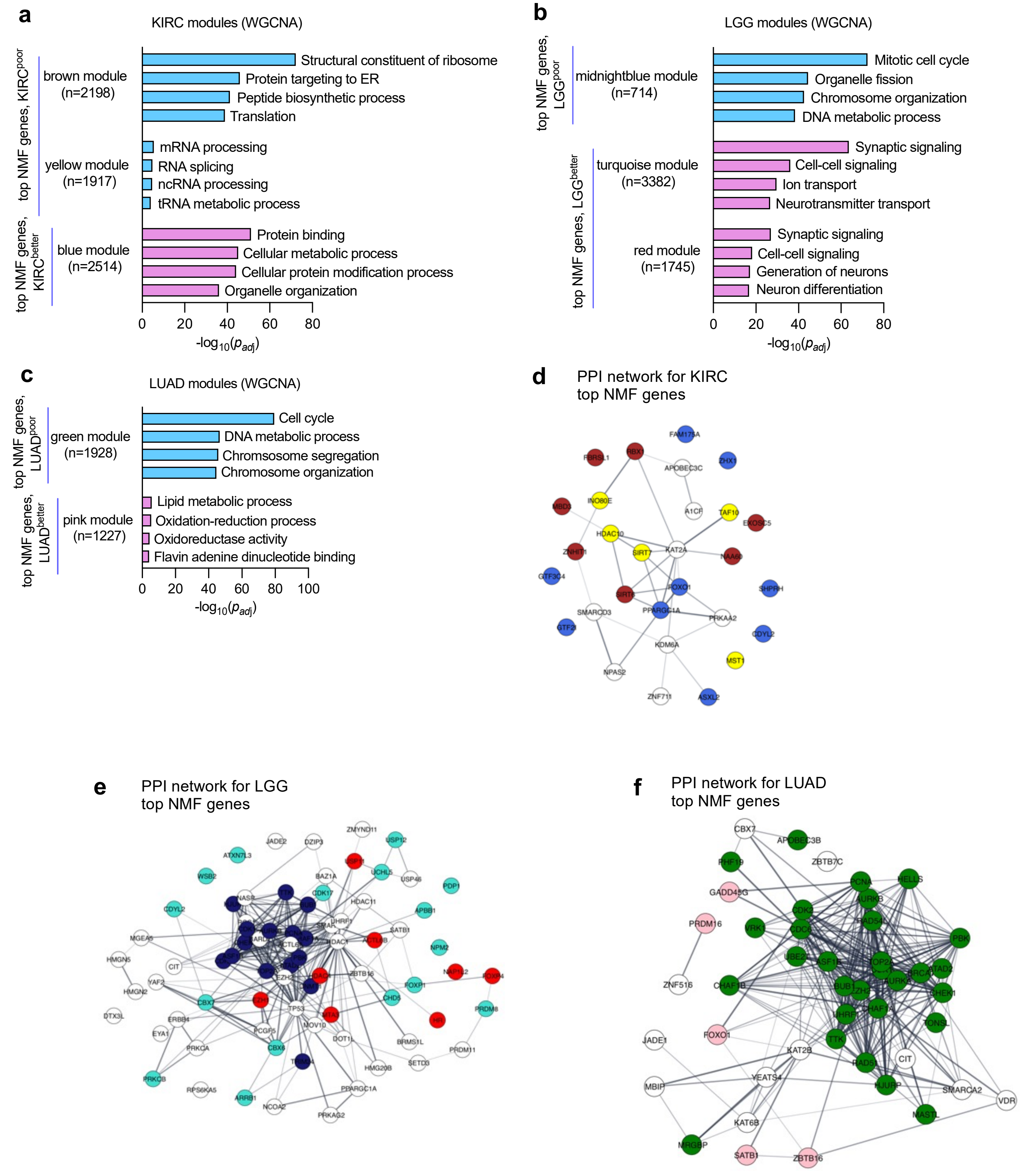

Supplementary Fig. 9 GO analysis of the WGCNA modules and PPI networks generated from the top NMF genes for the five-cancer group. Related to Fig. 3. **a-c** Significantly enriched GO terms for the WGCNA-derived modules that contain the top NMF genes for KIRC (**a**), LGG (**b**), and LUAD (**c**). **d-f** PPI networks formed from the encoded proteins of the top NMF genes for KIRC (**d**), LGG (**e**), and LUAD (**f**). See the Figure 3 legend for a detailed description of the generation of these plots.

Supplementary Fig. 10

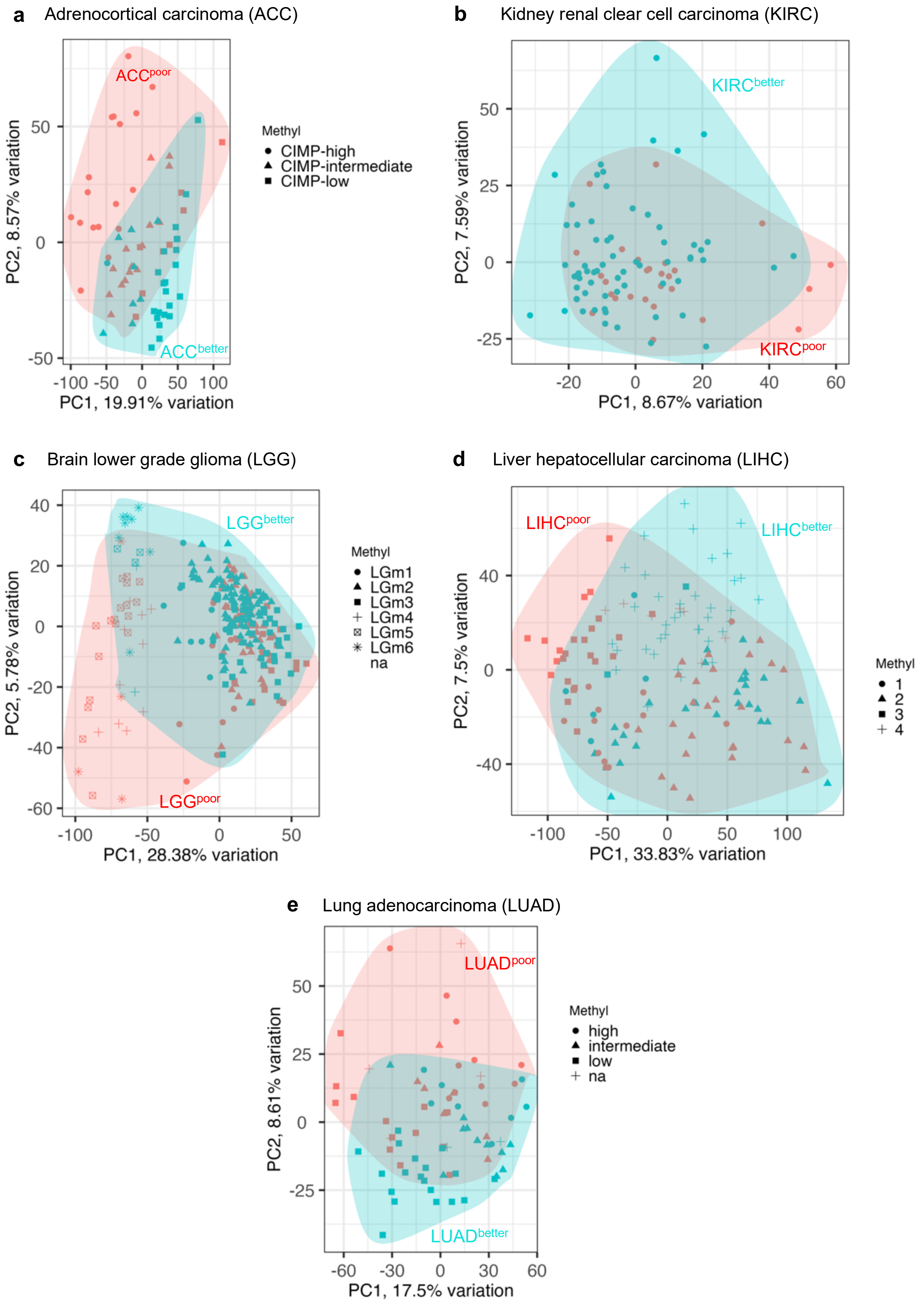

Supplementary Fig. 10 Low-dimensional representation based on DNA methylation patterns of the patient tumors included in the two epifactor expression–based NMF clusters. Related to Supplementary Fig. 11. **a-e** PCA plots based on array-based DNA methylation patterns for the patient tumors in the two NMF epifactor expression–derived clusters for ACC (**a**), KIRC (**b**), LGG (**c**), LIHC (**d**), and LUAD (**e**). The patient clusters denoted as poor (red) and better (cyan) are associated with worse and better outcomes, respectively. The different shapes of the plotted points represent the known DNA methylation–based subtypes from TCGA. The percent variance explained by PC1 (x-axis) and PC2 (y-axis) are shown. For KIRC, information about the DNA methylation subtypes is not available.

Supplementary Fig. 11

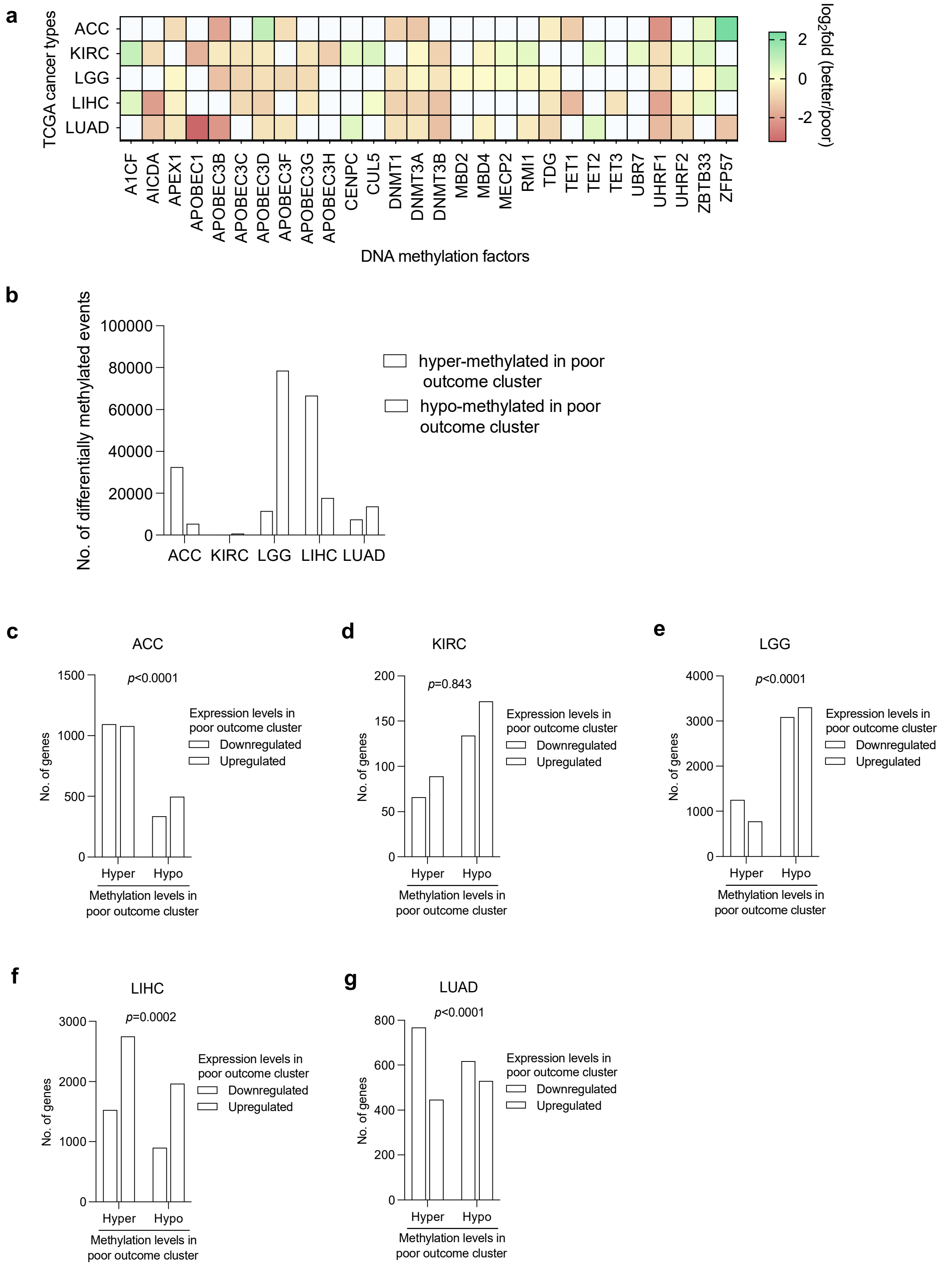

Supplementary Fig. 11 DNA methylation differences between the epifactor expression–based NMF clusters and their impact on gene regulation. Related to Supplementary Fig. 10**.** **a** Heatmap showing the differential expression of epifactors related to DNA methylation between the two epifactor expression–based clusters for the five-cancer group. Red and green represent higher expression in the poor outcome and better outcome cluster, respectively. **b** Bar plot showing the number and type of differentially methylated events for the two epifactor expression–based clusters. **c-g** Plots of the number of genes that are upregulated and downregulated in the poor outcome cluster among the genes that are hypomethylated or hypermethylated in the same cluster for ACC (**c**), KIRC (**d**), LGG (**e**), LIHC (**f**), and LUAD (**g**). The significance values (*p*) were determined using the two-tailed Fisher's exact test.

Supplementary Fig. 12

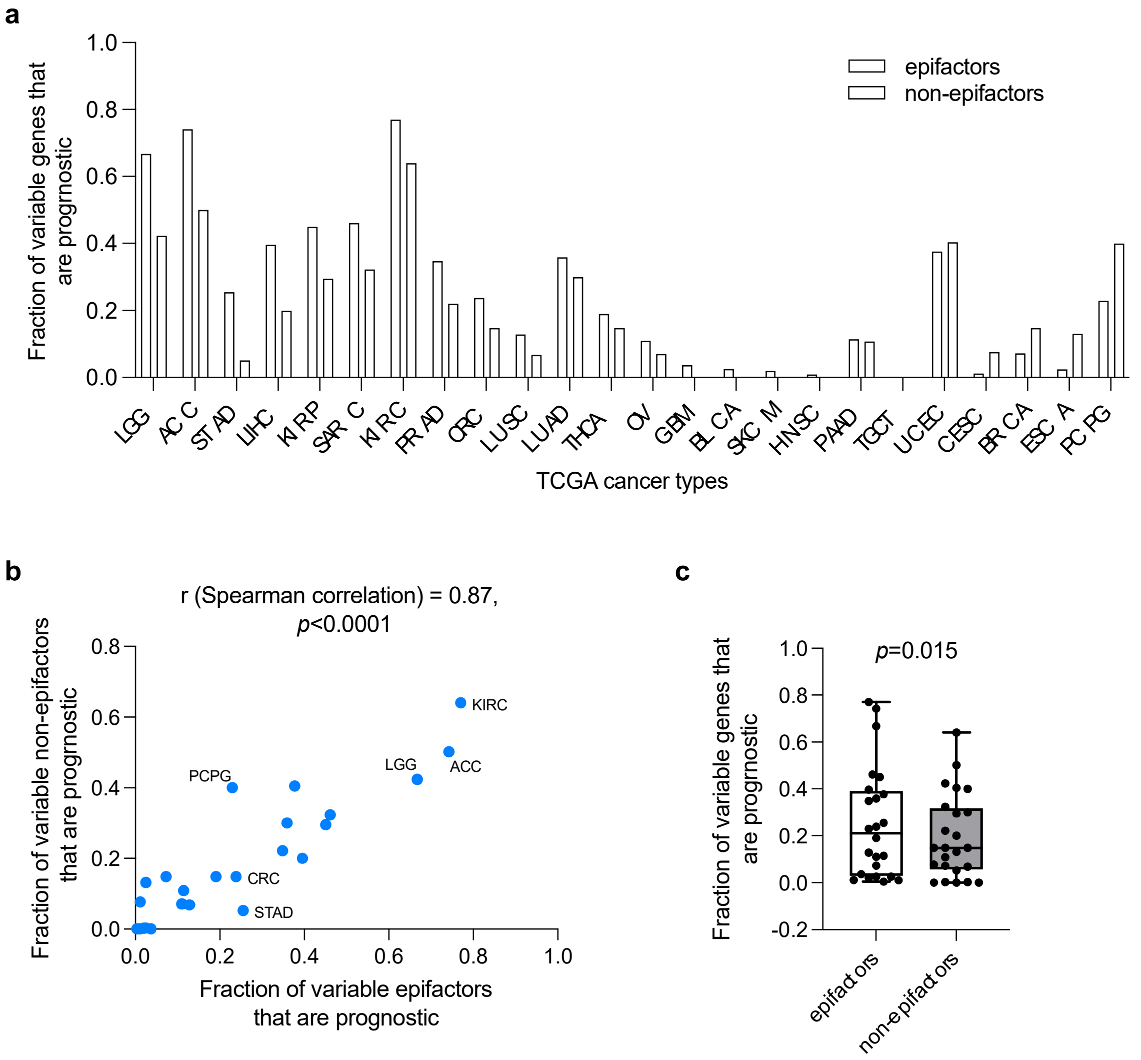

Supplementary Fig. 12 Comparison of the prognostic capacity of epifactors and non-epifactor genes. Related to Fig. 4. a Bar plot comparing the fraction of variable genes that are prognostic for epifactor and non-epifactor genes. The comparison was performed using the same parameters for both groups of genes across the 24 cancer types. **b** Correlation between the fractions of variable genes that are prognostic for epifactor and non-epifactor genes across the tumor types. The correlation coefficient (r, Spearman method) and significance of correlation (*p*, two-tailed) are shown. Each point represents one cancer type. **c** Box plot comparing the fractions of variable prognostic genes that are prognostic for epifactor and non-epifactor genes. The whiskers of the box plots indicate minimum and maximum values. The significance value (*p*) was calculated using two-tailed Wilcoxon matched-pairs signed rank test.

Supplementary Fig. 13

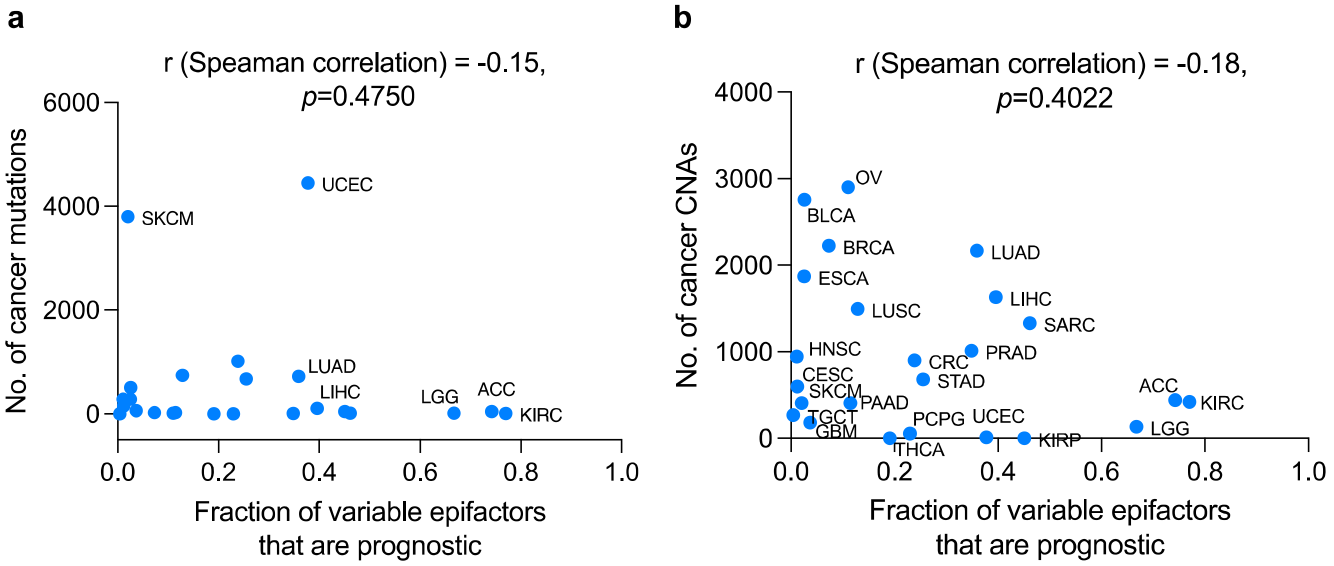

Supplementary Fig. 13 Relationship between the extent of genetic changes and the fraction of prognostic epifactors in a cancer type. **Related to Fig. 4.** **a-b** Correlation between the fractions of variable epifactors that are prognostic and the number of mutations (**a**) or the number of CNAs (**b**) among the 24 cancer types. The correlation coefficient (r, Spearman method) and significance of correlation (*p*, two-tailed) are also shown. Each point represents one cancer type. Only mutations and CNVs with frequencies greater than 10% in a cancer type were included.

Supplementary Fig. 14

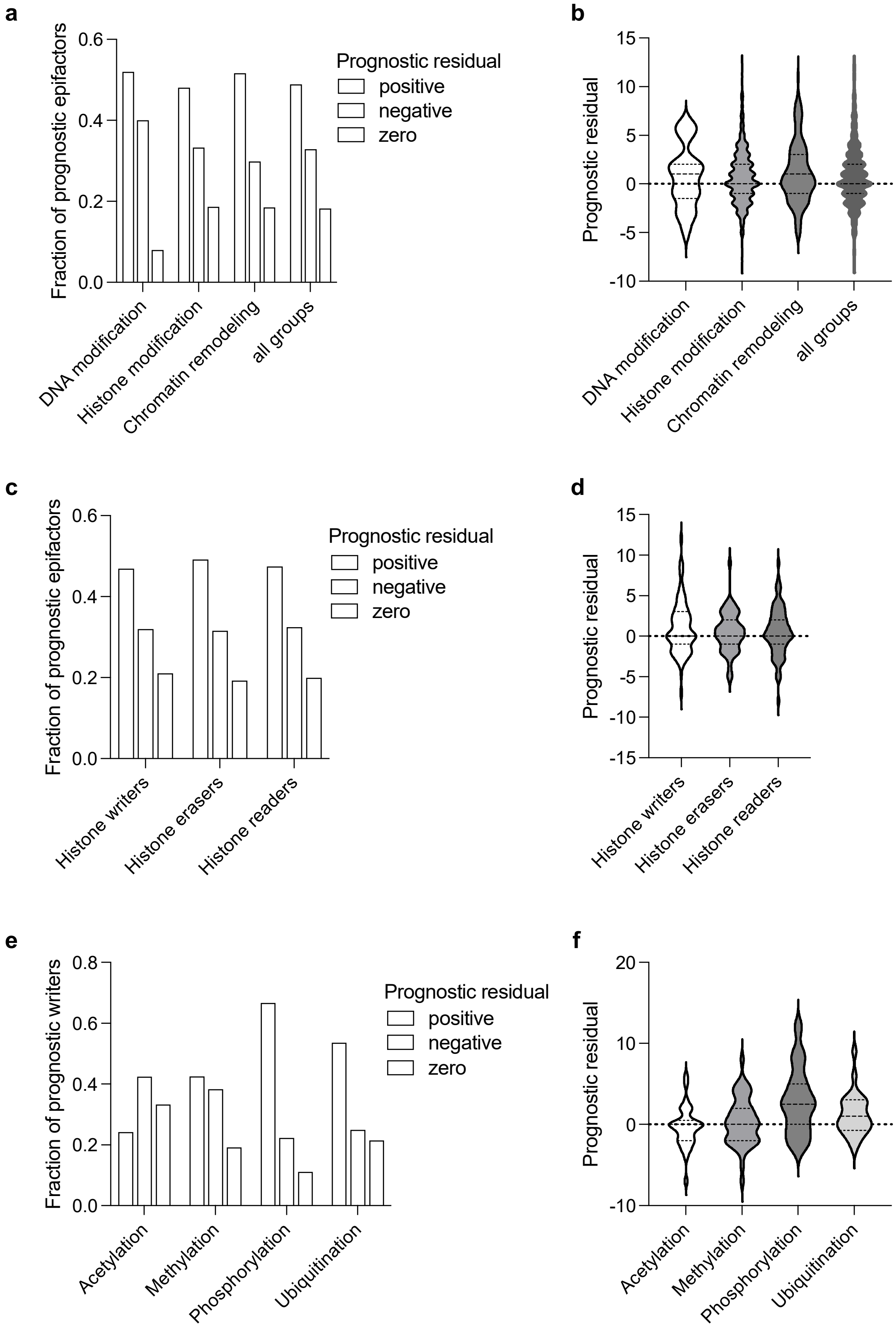

Supplementary Fig. 14 Prognostic residuals for the prognostic epifactors representing different functional groups. Related to Fig. 4. **a,c,e** Bar plots (left) comparing the fractions of prognostic epifactors with different levels of prognostic residuals (the number of cancer types in which high expression is associated with a poor outcome minus the number of cancer types in which low expression is associated with a poor outcome)for three major functional groups (**a**), histone writers/erasers/readers (**c**), and different kinds of histone writers (**e**). **b,d,f** Violin plots (right) comparing the distribution of prognostic residuals for the corresponding groups included in the bar plots on the left.

Supplementary Fig. 15

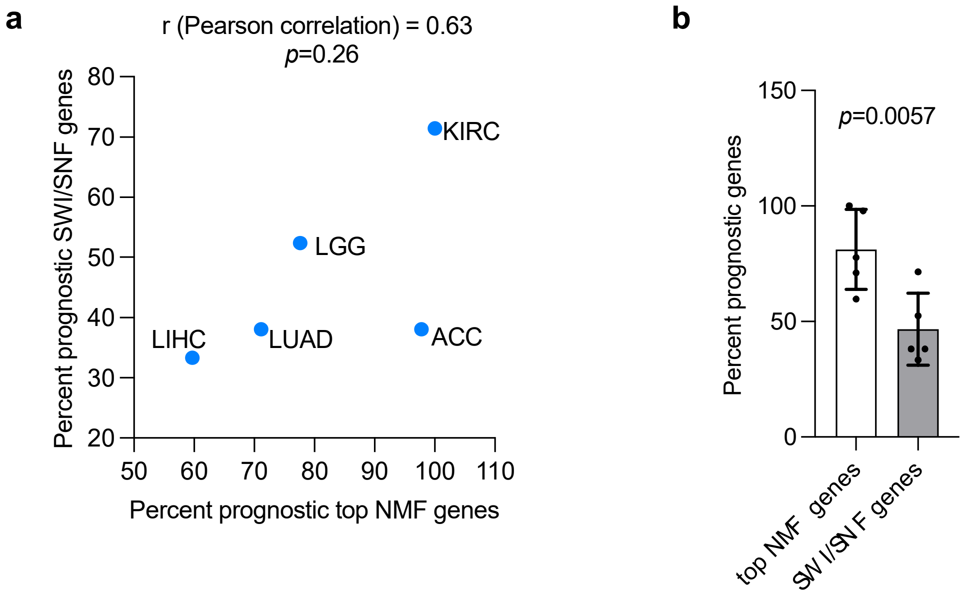

Supplementary Fig. 15 Comparison of prognostic efficacy of top NMF vs. SWI/SNF genes. **Related to Fig. 4.** **a** Percent prognostic genes for top NMF and SWI/SNF epifactors for the five cancer types. The correlation coefficient (r, Spearman method) and significance of correlation (*p*, two-tailed) are also shown. **b** Bar graph of the fraction of prognostic genes among the five cancer types for top NMF genes and SWI/SNF genes. The *p* value was calculated using the two-tailed paired t-test. Mean and standard deviation are shown.

Supplementary Fig. 16

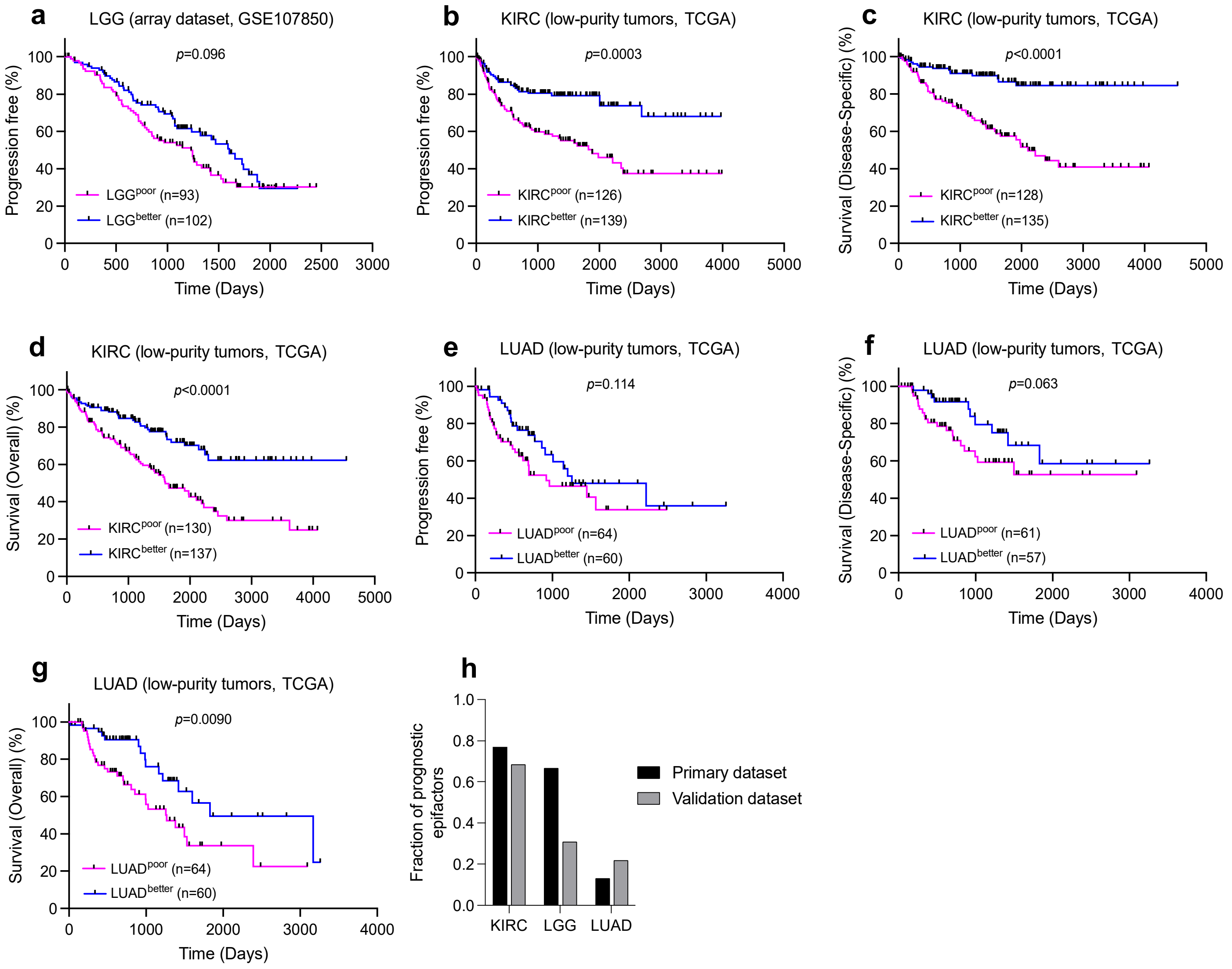

Supplementary Fig. 16 Clinical outcome differences between the poor outcome and better outcome patient groups and prognostic epifactors for the validation cohorts for LGG, KIRC, and LUAD. **Related to Supplementary Data 9.** **a-g** Kaplan-Meir plots comparing the poor outcome and better outcome groups for the validation cohort for LGG (**a**), KIRC (**b-d**), and LUAD (**e-g**). *p* values were obtained from the log-rank Mantel-Cox test. The number of patients (n) included in each group is shown. The assignment of patient tumors in the validation cohort to the poor outcome or better outcome group was based on the combined expression score of the group-specific top NMF epifactor markers derived from the analysis of the primary dataset (see Supplementary Fig. 4). **h** Bar plot comparing the fractions of variable epifactors that are prognostic for the primary and validation datasets for KIRC, LGG, and LUAD. For KIRC and LGG, the prognostic efficacy of epifactors is based on the PFI metric, while for LUAD, overall survival is used.

Supplementary Fig. 17

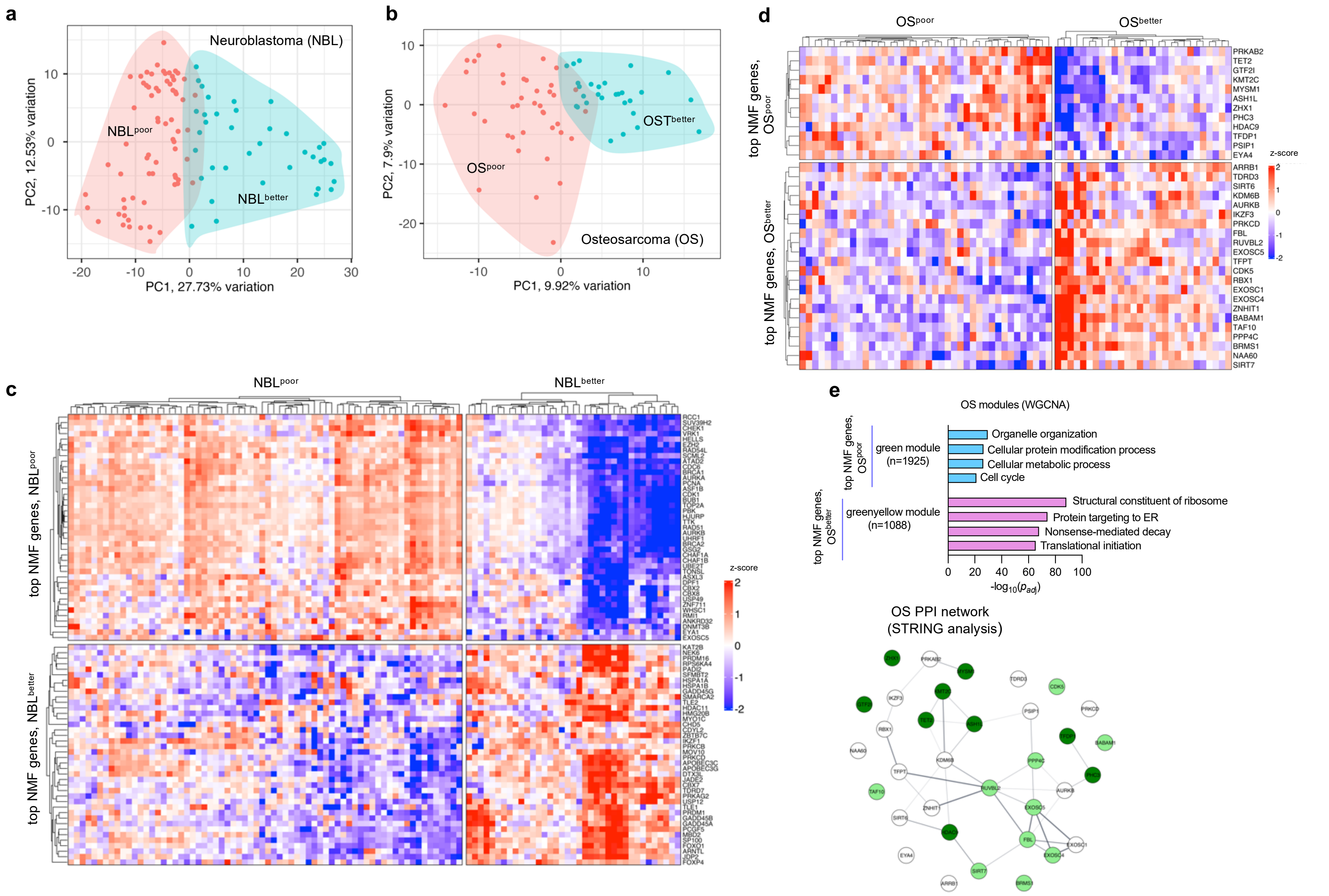

Supplementary Fig. 17 Signature epifactor genes and GO terms related to the clinically distinct epifactor expression–based clusters of NBL and OS. Related to Fig. 6. **a** and **b** PCA plots for the two epifactor expression–based clusters for NBL (**a**) and OS (**b**). **c** and **d** Heatmaps showing the expression patterns for the top NMF genes across the two clusters for NBL (**c**) and OS (**d**). **e** Upper panel: WGCNA-derived gene modules containing the top NMF genes for OS and the GO terms associated with these modules. The top NMF genes were upregulated in either the poor outcome or the better outcome OS cluster. Lower panel: PPI network formed by the encoded proteins of the top NMF genes of OS and color-coded according to the gene's WGCNA module membership.
