# Supplementary material for: Pan-cancer landscape of epigenetic factor expression predicts tumor outcome
Source: Commun Biol. 2023 Nov 16;6:1138. doi: 10.1038/s42003-023-05459-w (PMC10654613; doi:10.1038/s42003-023-05459-w)
Supplement: Supplementary file 2 — Description of Additional Supplementary Files [file 42003_2023_5459_MOESM2_ESM.pdf]

Description of Additional Supplementary Files

File name: Supplementary Data 1
Description: Patient selection for the study.

File name: Supplementary Data 2
Description: Information about the 720 epifactor genes used in this study and intersection with COSMIC/OncoKb and other databases.

File name: Supplementary Data 3
Description: Patient assignment into NMF clusters for 24 cancer types.

File name: Supplementary Data 4
Description: Top NMF genes for the 24 cancer types, and mutation and CNV analysis for the NMF clusters corresponding to the five-cancer group (ACC, KIRC, LGG, LIHC, and LUAD).

File name: Supplementary Data 5
Description: Immunologic and TCGA subtype information for the five-cancer group (ACC, KIRC, LGG, LIRC, LUAD).

File name: Supplementary Data 6
Description: WGCNA analysis for the five-cancer group (ACC, KIRC, LGG, LIRC, LUAD) and the enrichment metrics for the PPI networks.

File name: Supplementary Data 7
Description: DESeq2 and differential methylation analysis of the epigenetic factor–based clusters for the five-cancer group (ACC, KIRC, LGG, LIHC, LUAD).

File name: Supplementary Data 8
Description: Prognostic epigenetic factor genes (PFI) for the 24 cancer types before and after the meta-PCNA correction.

File name: Supplementary Data 9
Description: Ranked features for the 5-cancer and 4-cancer machine learning models and validation cohort analysis.

File name: Supplementary Data 10
Description: Patient selection criteria, cluster assignments, top NMF genes, and WGCNA analysis for the pediatric cancer types.

File name: Supplementary Data 11
Description: Prognostic epigenetic factor genes (overall survival) for pediatric and adult cancer types.

File name: Supplementary Data 12
Description: Source data behind graphs in the figures.

File name: Supplementary Data 13
Description: List of software packages used for this study.
